# Metasynthesis of Youth Suicidal Behaviours: Perspectives of Youth, Parents, and Health Care Professionals

**DOI:** 10.1371/journal.pone.0127359

**Published:** 2015-05-22

**Authors:** Jonathan Lachal, Massimiliano Orri, Jordan Sibeoni, Marie Rose Moro, Anne Revah-Levy

**Affiliations:** 1 AP-HP, Hôpital Cochin, Maison de Solenn, Paris, France; 2 Université Paris Descartes, Sorbonne Paris Cité, Paris, France; 3 INSERM, U1178, Paris, France; 4 Université Paris Sud-Paris 11, Paris, France; 5 Argenteuil Hospital Centre, Centre de Soins Psychothérapeutiques de Transition pour Adolescents, Argenteuil, France; Örebro University, SWEDEN

## Abstract

**Background:**

Youth suicide is a major public health issue throughout the world. Numerous theoretical models have been proposed to improve our understanding of suicidal behaviours, but medical science has struggled to integrate all the complex aspects of this question. The aim of this review is to synthesise the views of suicidal adolescents and young adults, their parents, and their healthcare professionals on the topics of suicidal behaviour and management of those who have attempted suicide, in order to propose new pathways of care, closer to the issues and expectations of each group.

**Methods and Findings:**

This systematic review of qualitative studies — Medline, PsycInfo, Embase, CINAHL, and SSCI from 1990 to 2014 — concerning suicide attempts by young people used thematic synthesis to develop categories inductively from the themes identified in the studies. The synthesis included 44 studies from 16 countries: 31 interviewed the youth, 7 their parents, and 6 the healthcare professionals. The results are organised around three superordinate themes: the individual experience, that is, the individual burden and suffering related to suicide attempts in all three groups; the relational experience, which describes the importance of relationships with others at all stages of the process of suicidal behaviour; and the social and cultural experience, or how the group and society accept or reject young people in distress and their families and how that affects the suicidal process and its management.

**Conclusion:**

The violence of the message of a suicidal act and the fears associated with death lead to incomprehension and interfere with the capacity for empathy of both family members and professionals. The issue in treatment is to be able to witness this violence so that the patient feels understood and heard, and thus to limit recurrences.

## Introduction

Suicide and attempted suicide are a major public health issue in Europe and throughout the world [1]. Youth—that is, adolescents and young adults, aged 15 to 29 years, and also referred to here as young people—are particularly at risk of suicidal behaviours: suicide is the second leading cause of death among this age group [1], and the rate of suicide attempts is estimated to be 10 to 20 times higher than that of completed suicides [2,3]. Worldwide, there are officially around 164 000 deaths by suicide annually among those younger than 25 years [4] and the sex ratio ranges from about 2 to 6 young men for every young woman. Distribution at the international level is heterogeneous, with prevalence higher in Eastern Europe, lower in Central and South America, and intermediate in the USA, Western Europe, and Asia. The rates in Africa are generally unknown [5–7].

In numerous Western countries, the incidence of suicidal behaviours among young people increased considerably from the beginning of the 20th century into the 1990s [7,8], when large-scale campaigns of prevention and the introduction of antidepressant treatments resulted in a significant reduction in deaths from suicide [9–12]. Although these prevention campaigns are ongoing [13], recent trends in many countries show that the prevalence rates of suicidal attempts have stopped falling; they are either becoming stable or starting to rise again [5,14,15].

Numerous theoretical models have been proposed to improve our understanding of suicide [3,5,10,16–19], but medical science has struggled to integrate all the complex aspects of this question at the interface of medical, sociological, anthropological, cultural, psychological, and philosophical issues [20]. Explanatory models are necessary, to allow us to think about suicide in a new and different way. Meta-synthesis is a useful and recognised tool that can help to understand complex medical questions [21–24]. It appears to be a tool of choice for apprehending questions about suicide and allows in-depth access to the perspectives of the different groups involved with young people who have attempted suicide.

We conducted a systematic review of the qualitative studies about suicidal behaviours in the medical literature and a meta-synthesis (through a thematic analysis) of 44 studies that interviewed youthful suicide attempters, their parents, and the healthcare professionals providing care to them [25,26]. We decided to include these three groups of participants because they are the main protagonists of the therapeutic relationship in this context. Our objective in conducting this review was to describe the experience of attempted suicide and its management as closely as possible from the perspective of each of these three groups, covering the issues and expectations of each, so that we can propose new pathways for thinking about and improving care.

## Methods

### Design

We used thematic synthesis [27]. Our procedure took place in four stages: designing the research, that is, defining the question, subjects, types of studies to include, and the protocol; the search for and selection of articles; and the analysis itself, in two separate stages, first a descriptive portion in which we determined and compared themes, and then an interpretive stage in which we constructed a descriptive schema of the phenomenon, original proposals that we then examined from the perspectives of theory, the literature, clinical practice, and care [25,27]. These steps increase both the possibilities for generalisation and the strength of these generalisations.[28] Our method is consistent with the ENTREQ statements [29]. (S1 Table)

Here are the six steps of our method:

1. Definition of the research question (summarised in the aims and objectives);
2. Identification and selection of studies;
3. Quality assessment of the selected studies;
4. Analysis of the papers, identification of themes, and translation of the themes across studies;
5. Generating analytical themes and structuring the synthesis
6. Writing the synthesis.

### Selection of Studies

We conducted a systematic search for qualitative studies specifically devoted to suicidal behaviours in young people (step 2). The QUALIGRAMH working group (Qualitative Group for Research in Adolescent Mental Health, INSERM U 669, Maison des Adolescents, Hôpital Cochin, Paris), composed of specialists in qualitative research and disorders of young people, defined the study criteria.

The papers were selected only if they met the following criteria:

- Used solely qualitative methodology.
- Specifically concerned suicidal behaviours in adolescents and young adults (referred to hereafter as youth or young people).
- Interviewed:
  - Young people who were suicidal, or who had attempted suicide in their youth, or
  - Parents of these youth, or
  - Medical professionals who provide care to suicidal youth.
- Were published in English or French between 1990 and May, 2014 (the period covering most of the qualitative articles about suicide).

Finally the following studies were excluded:

- Studies using quantitative or mixed methodologies;
- Studies in the general population exploring prevention of suicide or social representations of suicide in adolescents and young adults;
- Studies concerning solely deliberate self-harm or non-suicidal self-injury.

The study was conducted from January to May 2014. An initial search identified a selection of papers, from which we collected keywords. Based on this selection as well as on existing literature reviews about suicide [30–32], the research group drew up a list of keywords, a mix of free-text terms and thesaurus terms related to suicidal behaviours, youth, and qualitative research [33,34] and compiled a list of databases indexing qualitative studies in the fields of medicine, sociology, and psychology [25,35]. We performed our search on July 1, 2013 (and updated it on May 31, 2014) (Table 1 and S2 Table).

**Table 1:** Web searches—January 1, 1990 to July 1, 2013 (updated on May 31, 2014).

Databases	Free-text terms keywords	Thesaurus terms keywords	References
MEDLINE	26	19	194
CINAHL	26	27	593
PsycInfo	26	30	169
Embase	26	21	266
SSCI	26	0	582
**TOTAL**	**-**	**-**	**1804**

**Fig 1:**
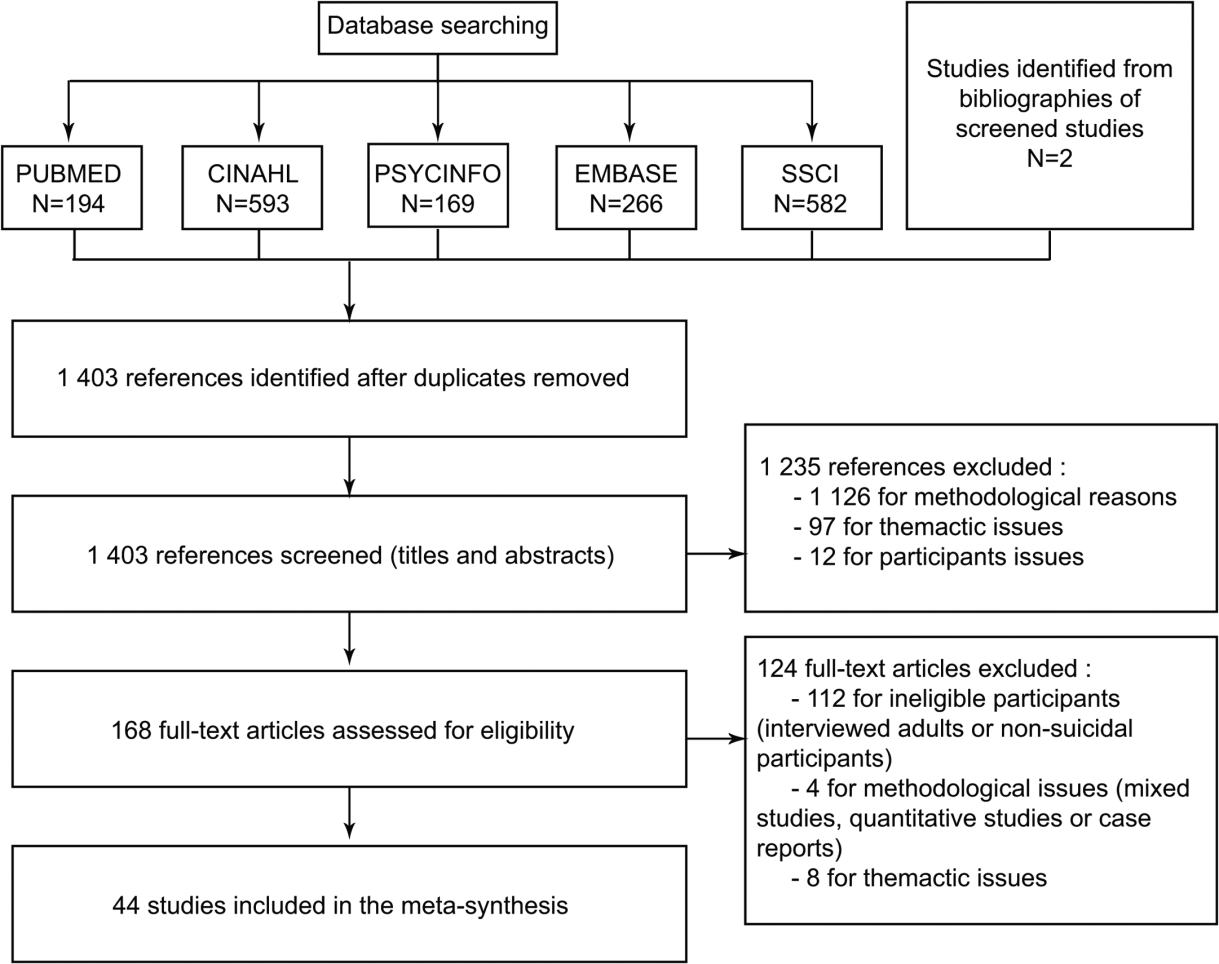
Flowchart for selecting studies.

In all, we obtained 1804 references, 1403 of which remained after removal of duplicates (Fig 1). Two authors (JL and MO) screened all titles and abstracts, according to the relevance of their theme and methodology. If the abstract was not sufficient, the full text was read. Disagreements were resolved during working group meetings. For example, we initially included all studies concerning deliberate self-harm or non-suicidal self-injury in our selection. After discussions and literature review [3], we decided to exclude all these papers because we do not think that the issues of suicide and self-harm are identical: the question of death is posed differently for these two groups of subjects. Another issue was whether or not to include mixed studies, given that the best way of dealing with mixed methods remains unclear [25]. After consulting these papers, we concluded that they did not contribute to our thematic framework and decided to exclude them from the analysis. Full texts of potentially relevant papers were then examined, and a second selection was performed (the papers excluded at this point and the reasons for their exclusion are listed in S1 File). After removal of studies that did not meet the criteria defined above, 42 papers remained. Scanning the reference lists for more potentially relevant papers provided 2 more papers. In all, the review finally included 44 studies, around 2.5% of the papers screened. This rate is consistent with the findings of other such meta-syntheses [25,33,34].

### Assessment of Paper Quality

The evaluation of quality (step 3) is necessary to enable discussion of the studies and to ascertain the value and integrity of the data used. The working group chose a tool widely used for medical meta-syntheses, the Critical Appraisal Skills Programme (CASP) [32,36]. Two researchers (JL and MO) independently performed the evaluation, and the working group reached a consensus about it. Table 2 summarises the quality evaluation criteria.

**Table 2:** Evaluation of the quality of the studies according to the Critical Appraisal Skill Programme (CASP).

Criteria	Totally Met^1^	Partially Met^1^	Not Met^1^
1. Was there a clear statement of the aims of the research?	41	3	0
2. Is a qualitative methodology appropriate?	41	3	0
3. Was the research design appropriate to address the aims of the research?	39	5	0
4. Was the recruitment strategy appropriate to the aims of the research?	31	10	3
5. Were the data collected in a way that addressed the research issue?	37	6	1
6. Has the relationship between researcher and participants been adequately considered?	25	10	9
7. Have ethical issues been taken into consideration?	36	1	7
8. Was the data analysis sufficiently rigorous?	24	16	4
9. Is there a clear statement of findings?	28	9	7
10. How valuable is the research?	29	15	0

^1^ Number of studies.

### Data Analysis

We followed the procedure described by Thomas and Harden (2008) to analyse the data [27]. The analysis (step 4) included a careful reading of the titles, abstracts, and complete papers as well as repeated rereadings. One researcher (JL) extracted the formal characteristics of the studies, while he and two others (ARL and MO) independently extracted and analysed the data, which were then compared during meetings. We used thematic analysis to develop categories inductively from the first-order themes identified in these studies. Afterwards, the translation work involved comparing themes across papers to match themes from one paper with those from another and ensure that each key theme captured similar themes from different papers. Finally, we ordered these translation results into a framework containing three superordinate themes (step 5). This step is more interpretive, since the group decided to organize the themes through a more conceptual line of argument. The last step consisted in expressing the synthesis in a useful form (step 6). This process led not to a summary of the different studies included, but to an interpretation of the papers, described in the discussion. Research meetings were held regularly to discuss the results obtained. The triangulation of sources—meta-synthesis does this by definition—and the triangulation of reviewers made possible a high level of rigour in the results [37,38].

## Results: Description of the Studies

### Presentation of the Included Studies

In all, we examined 31 studies that questioned young suicide attempters, by semi-structured interviews, free interviews, focus groups, internet forums or chat rooms, or questionnaires. Six studies questioned healthcare professionals who provide care to suicidal youth; these used semi-structured interviews and focus groups to collect date from doctors, nurses, psychiatrists, psychology students, social workers, and counsellors. Finally, seven studies questioned the parents of suicidal youth, in structured, semi-structured, or unstructured interviews. Studies come from 16 different countries: 16 from North and Central America (Canada, Nicaragua, and the USA), 13 from Europe (Italy, Norway, Sweden, and the United Kingdom), 3 from Africa (Ghana and South Africa), 6 from Asia (Hong Kong, Israel, Iran, Vietnam and the Republic of Korea), and 6 from Oceania (Australia and New Zealand). Tables 3, 4 and 5 detail the characteristics of each study.

**Table 3:** Main characteristics of the studies (Studies interviewing young people who have attempted suicide).

**Study**	Aims	Country	Nb^1^	Age^1^	Data coll.^2^	Analysis method
[81]	To identify different contexts in which young people harm themselves and propose a theory generated from the data that might link family circumstances, suicidal cognition, suicidal phantasies and the nature of the self-harming act itself.	UK	23	9–16	SSI, Q	Grounded theory
[62]	To explore the accounts of young adults who engage in self-harming and suicidal behaviours and use websites dedicated to these issues, in order to develop a broader understanding of these websites and to identify potential implications for future research.	UK	10	18–30	SSI	Foucauldian discourse analysis
[63]	To develop an understanding of non-fatal suicidal behaviour (NFSB) from the perspective of individuals of a relatively homogenous group of respondents who shared characteristics which placed them at a particularly high risk for NFSB (i.e., female adolescents of Indian origin)	South Africa	10	14–17	SSI	Thematic analysis
[43]	To explore young people’s transitions towards resistance against future suicidal behaviours.	New Zealand	27	15–24	SSI	Discourse analysis
[44]	To highlight the complexities evident in young people’s engagement with discourses of depression and mental ill-health, which constitute a dominant part of the construction of their suicidal behaviour, and examine the ways in which they negotiate and renegotiate relationships with dominant constructions of mental ill-health that combine youth, suicidal behaviour, deviance, and psychopathology.	New Zealand	30	16–25	SSI	Discourse analysis
[64]	To develop a grounded theory of recovery from the perspective of young adults with a history of repeated suicide-related behaviour who completed at least one cycle of a specific treatment intervention: psychosocial/psychoeducational intervention for people with recurrent suicide attempts (PISA).	Canada	16	18–25	SSI	Grounded theory
[45]	To examine how adolescents who have overcome suicidal thoughts and behaviours perceive the role of attachment relationships in the process of becoming suicidal.	Canada	50	13–26	SSI	Grounded theory
[46]	To explore how adolescents perceive attachment relationships as helping them overcome suicidality.	Canada	50	21,9	SSI	Grounded theory
[65]	To capture adolescents’ own perspectives regarding the factors implicated in their psychological distress, to better understand how these youth conceive the causes of their depression/suicidal ideation, and to focus on the quality of the relationships between suicidal adolescents and their parents and the role these relationships played in the adolescents’ psychological distress or resilience.	Israel	10	15–19	SSI	Consensual qualitative research
[50]	To identify common themes in answer to the question, "What was the experience of young adults who felt suicidal, made the decision, and attempted to end their lives?"	Canada	5	24–27	SSI	Phenomenology
[48]	To illustrate the role 3 major factors played in one teenager's experience of becoming and overcoming being suicidal: mental processes, cognitive development, identity formation, and autonomy-seeking.	Canada	1	20	SSI	Grounded theory
[49]	To develop an understanding of how adolescents and emerging adults experience and respond to emotions from the subjective perspective of previously suicidal participants.	Canada	50	15–27	SSI	Grounded theory
[47]	To develop an understanding of how adolescents overcome suicidality from the subjective perspective of previously suicidal female participants, using a resilience framework to conceptualize the process.	Canada	13	17–26	SSI	Grounded theory
[51]	To gain further insight into the factors that may buffer L/B/G youth from suicidality and present a model integrating the known risk factors, with the resiliency factors that emerge.	New Zealand	8	18–25	SSI	Grounded theory
[83]	To explore the experiences of adolescents seeking help online for suicidality, focusing on online helper therapy as a key finding.	Canada	10	Ado	M	Content analysis
[52]	To explore perceived causes, and discover triggers and processes leading to suicidal behaviour among adolescent girls in Leo´n, Nicaragua, and to develop a tentative conceptual model to understand the pathways to suicidal behaviour.	Nicaragua	8	15–19	SSI	Grounded theory and Content analysis
[53]	To explore the thoughts of students who had experienced suicidal ideation but had not attempted suicide, and to identify specific themes of suicidal ideation among college students in South Korea.	Republic of Korea	134	18–28	Q	Qualitative content analysis
[66]	To learn first-hand from young men about the context of their suicidal behaviour and to use this contextual perspective as a basis for thinking about service delivery and clinical care.	UK	36	<30	SSI	Grounded theory
[54]	To illuminate the sociocultural contexts of attempting suicide among Iranian youth.	Iran	25	14–17	FI	Thematic analysis
[55]	To identify the factors that contribute to suicide, to review the signs and characteristics associated with these factors, to interview Mexican-American students in special education programs for emotional and behavioural disorders who exhibited various characteristics of suicidal thoughts and/or have attempted suicide, to explore effective prevention programs, and to provide suggestions for school personnel.	USA	8	13–18	SSI	Phenomenology
[67]	To explore and understand the pathways leading to attempted suicide among young men in Nicaragua, and to investigate the interplay between structural conditions and individual coping strategies, as well as to achieve an in-depth understanding of what triggers suicidal behaviour in young men.	Nicaragua	12	15–24	SSI	Grounded theory
[56]	To explore the perspective of adolescents who have directly engaged in suicidal acts (in either single or repeated suicide attempts).	Italy	16	17–25	SSI	IPA
[68]	To investigate what individuals who were suicidal between the ages of 13 to 18 report as being helpful in psychotherapy to overcome suicidal thoughts, feelings and behaviours, in order to increase our understanding of helpful aspects of psychotherapy in previously suicidal adolescents.	Canada	37	21,8	SSI	Multidimensional scaling and clustering analysis methods
[69]	To analyse the interactions between the users of a non-professionally run deliberate self-harm message board, focusing on the function of the message board as manifested in users’ interactions.	UK	174	Adolescents	M	IPA
[57]	To explore the possible reasons for the participants’ suicide attempt, focusing on demographic characteristics, psychosocial factors, and environmental and cultural factors.	South Africa	14	13–20	SSI	Thematic analysis
[70]	To examine how patients who had previously presented to hospital after an episode of deliberate self-poisoning, but who had not harmed themselves in the past two years, discussed their self-harming behaviour and the health services they received at the time, and to identify how patients accounted for this resolution.	UK	9	16–25	SSI	Grounded theory and narrative analysis
[58]	To explore in depth how adolescents with suicidal ideation perceived their family, school, and peer relationships, including as support systems, and to shed light on the operation of school guidance, together with parental and peer support in maintaining adolescent psychological health and in preventing suicide.	Hong-Kong	13	11–18	SSI	Data display, data interpretation, and drawing conclusions
[59]	To explore further the qualitative responses of hospitalized, suicidal adolescents after the AFI intervention in order to determine whether participating in the AFI intervention was meaningful to the participants.	USA	11	13–18	SSI	Phenomenology
[60]	To explore the suicidal process, suicidal communication and psychosocial situation of young suicide attempters in a rural community in Hanoï	Vietnam	19	15–24	SSI	Thematic analysis
[84]	To highlight the sociocultural themes that affect suicide attempts by Korean adolescents, contributing to a cross-cultural perspective that informs scholars in other nations of multiple realities, cultural awareness, and complex cultural interrelationships.	Republic of Korea	1	16	LH	Life history
[61]	To examine the conditions in which suicide attempts occur among young Latinas, how they experience the circumstances that led to the attempt, and what they say precipitated their suicide attempts and what triggered the act.	USA	27	11–19	SSI	Grounded theory

^*1*^ *Number and Age of participants;*

^*2*^ *Data collection; FI*: *free interviews; LH*: *life history; M*: *message boards; Q*: *questionnaires; SSI*: *semi-structured interviews; UK*: *United Kingdom; IPA*: *Interpretative phenomenological analysis*.

**Table 4:** Main characteristics of the studies (Studies interviewing parents).

**Study**	Aims	Country	Nb^1^	Age^1^	Data coll.^2^	Analysis method
[71]	To describe the experience of mothers living with suicidal adolescents.	Canada	6	-	FI	Phenomenology
[72]	To interview surviving family members who had lost a teenager by suicide to increase the understanding of the circumstances in which these families live.	Sweden	10	-	FI	Grounded theory
[74]	To provide a qualitative understanding of the experiences of preparedness for the suicide death of a young adult son or daughter from the perspective of parents	Australia	22	-	SSI	Narrative analysis
[73]	To explore parents’ experiences following the suicide death of their young adult child.	Australia	22	-	SSI	Narrative analysis
[75]	To explore the kind of experiences that suicidees had when seeking support from healthcare services in the period leading up to their death, as perceived by close family and friends.	Australia	15	-	SSI	Grounded theory
[77]	To understand suicide from the perspective of those who knew the deceased and were caught up in events surrounding the death.	UK	14	-	SSI	Narrative analysis
[76]	To build a tentative conceptual model, grounded in the parents’ views, of the process behind suicide in boys.	Sweden	51	-	SI	Grounded theory

^*1*^ *Number and Age of participants;*

^*2*^ *Data collection; FI*: *free interviews; SSI*: *semi-structured interviews; SI*: *structured interview; UK*: *United Kingdom*.

**Table 5:** Main characteristics of the studies (Studies interviewing health professionals).

**Study**	Aims	Country	Nb^1^	Age^1^	Data coll.^2^	Analysis method
[42]	To explore the attitudes towards young people who engage in suicidal behaviour, among nurses, nursing lecturers, and doctors.	UK	8		SSI	Constant comparative method
[78]	To focus on suicidal behaviour in young people by exploring the perceptions of this phenomenon among nurses and doctors working in accident and emergency, paediatric medicine and child and adolescent mental health services.	UK	45	-	SSI	Grounded theory
[82]	To explore nurses’ and doctors’ perceptions of young people who engage in suicidal behaviour, using a social semiotic theory to build an interpretation of the meanings nurses and doctors assign in relation to this group of young people	UK	45	-	SSI	Grounded theory and Social semiotic
[85]	To examine the attitudes of psychology students toward suicidal behaviour to understand the meaning(s) they assign to the act, and to discuss the consequences for suicide prevention in Ghana.	Ghana	15	-	SSI	IPA
[80]	To explore how outpatient counsellors engage parents following a youth suicide assessment and to add to the literature of engaging parents in rural environments, specifically around the issue of gun safety and suicide prevention.	USA	24	-	FG	Inductive analysis
[79]	To develop knowledge about the significance of ASIST (Applied Suicide Intervention Skills Training) for Public Health Nurses’ (PHNs) practice.	Norway	16	-	FG	Qualitative content analysis

^*1*^ *Number and Age of participants;*

^*2*^ *Data collection; FG*: *focus groups; SSI*: *semi-structured interviews; UK*: *United Kingdom; IPA*: *Interpretative phenomenological analysis*.

### Quality Assessment

Our evaluation found that on the whole the quality of the studies was good (Table 2 and S3 Table). The ethical considerations were sometimes insufficient, and the description of the analytical method sometimes inadequately detailed. This flaw was most often explained by editorial constraints (maximum word lengths that were more appropriate for the presentation of quantitative than qualitative research).

No study was excluded from the analysis on the basis of this evaluation. The original authors of the meta-ethnographic approach report that poorer quality studies tend to contribute less to the synthesis [25,39–41]. Further, there is no consensus on the role of quality criteria and how they should be applied, in particular for systematic reviews (see also [25,38]).

## Results: Thematic Synthesis

The thematic analysis clearly showed three superordinate themes of experience. The first is individual experience, comprising three subthemes: *the experience of distress*, *self-control*, *and the parents’ impotence in the face of the suicide attempter’s distress*. The second superordinate theme is the relationship with others, including the subthemes: *changes in the relational distance*, *feelings of difference and rejection*, and *the experience of incomprehension*. The third main theme concerns the social and cultural aspects of the suicidal act. The themes that compose it are: *failure to fit in the group*, and *sociocultural facilitators and barriers to suicide and its management* (Tables 6, 7, 8 and 9).

**Table 6:** Themes identified in each study.

Study	Experience of distress	Self-control	Parental importence in the face of the young suicide attempters’ distress	Changes in the relational distance	Feelings of difference and rejection	The experience of incomprehension	Failure to fit into the group	Sociocultural facilitators and barriers to suicide and its management
[42]	**Y**			**Y**	**Y**	**Y**		**Y**
[78]			**Y**	**Y**	**Y**	**Y**		**Y**
[82]				**Y**		**Y**		**Y**
[81]				**Y**				
[62]		**Y**				**Y**		**Y**
[63]		**Y**		**Y**			**Y**	**Y**
[43]	**Y**			**Y**		**Y**	**Y**	
[44]	**Y**	**Y**					**Y**	
[64]		**Y**						
[45]	**Y**	**Y**		**Y**	**Y**		**Y**	
[46]	**Y**	**Y**		**Y**		**Y**		
[71]			**Y**	**Y**	**Y**	**Y**		**Y**
[65]		**Y**		**Y**	**Y**	**Y**	**Y**	**Y**
[50]	**Y**	**Y**		**Y**	**Y**	**Y**	**Y**	
[48]	**Y**	**Y**		**Y**			**Y**	
[49]	**Y**	**Y**		**Y**	**Y**	**Y**	**Y**	**Y**
[47]	**Y**	**Y**		**Y**		**Y**		
[51]		**Y**		**Y**	**Y**	**Y**	**Y**	**Y**
[83]				**Y**		**Y**		**Y**
[52]				**Y**	**Y**	**Y**	**Y**	**Y**
[53]	**Y**	**Y**		**Y**	**Y**	**Y**	**Y**	**Y**
[66]		**Y**		**Y**		**Y**	**Y**	**Y**
[54]	**Y**			**Y**	**Y**		**Y**	**Y**
[72]			**Y**	**Y**		**Y**	**Y**	**Y**
[74]			**Y**		**Y**	**Y**		
[73]			**Y**					**Y**
[55]	**Y**			**Y**	**Y**	**Y**	**Y**	
[67]		**Y**		**Y**	**Y**		**Y**	**Y**
[75]			**Y**					
[85]						**Y**		**Y**
[56]	**Y**	**Y**		**Y**	**Y**	**Y**	**Y**	
[77]			**Y**		**Y**	**Y**	**Y**	**Y**
[68]		**Y**		**Y**		**Y**		
[69]		**Y**		**Y**		**Y**		**Y**
[57]	**Y**			**Y**	**Y**	**Y**	**Y**	
[70]		**Y**		**Y**	**Y**	**Y**	**Y**	
[80]			**Y**	**Y**		**Y**		**Y**
[58]	**Y**			**Y**	**Y**	**Y**	**Y**	
[79]			**Y**	**Y**		**Y**		**Y**
[76]			**Y**	**Y**	**Y**	**Y**	**Y**	
[59]	**Y**	**Y**			**Y**	**Y**	**Y**	
[60]	**Y**			**Y**	**Y**	**Y**	**Y**	**Y**
[84]				**Y**	**Y**		**Y**	
[61]	**Y**	**Y**		**Y**	**Y**		**Y**	**Y**
TOTAL	18	21	10	35	24	32	26	24

**Table 7:** Quotations from participants and authors of primary studies to illustrate each theme of superordinate theme 1 (Individual experience).

Themes	Quotations from participants in primary studies	Interpretations of findings offered by authors
**The experience of distress**	Depressive symptoms: You’re going to school, you’re getting an education, but you’re depressed. [50]	Depressive symptoms: There is consistency in recognising the components we identify as despair. [49]
	Failure, defeat, self-disgust: I hated myself. [49]	Failure, defeat, self-disgust: She had a strong feeling of hatred toward herself; she saw herself as being fat, ugly, stupid, and blamed herself for difficulties in her life and in her family. [48]
	Getting better: I was really surprised at my will to live, to sort of keep going. [43]	Getting better: Many participants emphasised ‘thinking positively’ about their lives and concerns as a useful component of problem solving.[43]
**Self-control**	Impression of losing control: I felt I had no control over my life. Whatever I did didn’t matter. It was going to happen anyways. [49]	Impression of losing control: Thinking about suicide became a means of coping. [49]
	Regaining control: I became quite social after coming out. I suppose, yeah. . . I always just needed to constantly find that affirmation, it wasn’t just something that I could say okay I’ve had enough, you know. I constantly needed that every day. ... and it was also about my whole, my self-esteem and confidence just totally went up every time as well, and if I stopped or something they’d just go down again. [51]	Regaining control: Leon’s strategy similarly involved problem-focussed coping; he actively strengthened his social support network to secure self-esteem. [51]
**Helplessness of the parents in the face of the suicide attempters' distress**	The experience of parents: There was nothing in place for my other children. They were stuck in the middle; so was the whole family. You can’t give them [the other children] what they deserve because you are too wrapped up. [71]	The experience of parents: They experienced a loss of hope for an ending to the self-destructive behaviours, for a return of their formerly happy lives, for a bright and prosperous future for their children and themselves. [71]
	The therapists' experience of helplessness: I think it is more a feeling inside which you see and just mull over and then not worry about it but at the time it’s like well I have done this and that is all I can do. I can’t do any more. It is also on their part—you get frustrated. Because they just don’t take your help when you’re offering it—and you think well, ‘‘listen to me I am trying to help you I am trying to give you these opportunities but you don’t want them”. [78]	The therapists' experience of helplessness: Part of the feeling here is that whatever you [nurses and doctors] do, nothing seems to work. However, the core meaning is about suicidal behaviour being treated in a specialist setting which is used to ‘changing things’ with physical interventions. The meaning of frustration in this sense was linked to not being able to treat suicidal behaviour as if it were a physical illness. [78]

**Table 8:** Quotations from participants and authors of primary studies to illustrate each theme of superordinate theme 2 (Relational experience).

Themes	Quotations from participants in primary studies	Interpretations of findings offered by authors
**Changes in the relational distance**	Relational difficulties: My parents didn’t care. They didn’t want to help me or make things better. They just wanted to rag on me. . . that’s how everything seemed. And if nobody cared, why bother trying to live? In my mind I was alone. There was nobody. [45]	Relational difficulties: The unavailability of care and support created distant interpersonal relationships, feelings of loneliness and withdrawal. They became vulnerable to suicidal behaviours and, as they described, were unable to turn to others for comfort. [45]
	Suicide as a form of communication: It’s a powerful form of communication and the trick is what they are trying to communicate to us. [82]	Suicide as a form of communication: While it can be seen that suicidal behaviour is a communicative event, the meaning in the message was not always clear. [82]
	Rapprochement with family: My parents started treating me older and me and my dad would go out and have a coffee and then we'd be able to sit and talk and then slowly we got like this relationship. I'd go out more so then I wasn't always home with my mom and so we got along better too. [48]	Rapprochement with family: Changes in significant attachment relationships accompanied Marie's shift to resolving her suicidal state. [48]
	Need for attention: When I use the term `attention seeking' I don't use it in a derogatory way… I would suspect that there are a group of people who deliberately self-harm who will do it for. . . attention seeking primarily. [42]	Need for attention: The doctors in the study also showed they felt suicidal behaviour could be a way of `seeking attention', but that it occurred in specific groups. [42]
**Feelings of difference and rejection**	The adolescents had a feeling of difference and rejection: Nobody saw the likes of me when I came to this school. Everybody was tripped out because my pants had holes all the way down, and my hair was long, and everybody was just kind of like surprised to see someone like me. They [students] even gave me nicknames like warlock. They just gave me a whole bunch of them—let me see warlock, psycho, Freddie, Jason You know like the horror movie people? It was like that. When someone would dare me to do something, I'd do it. [55]	The adolescents had a feeling of difference and rejection: Social desirability may be important in the prediction of suicidal ideation and interest. [55]
	Parents feel rejected: Yeah, yeah, because he was just splitting the whole family, wasn’t he? He didn’t have any regard for anybody’s property. He would come and go as he pleased. [. . .] And we said to him, ‘Well, you don’t want to be part of this family. It’s obvious you don’t, because you wouldn’t be doing the things you’re doing’. So he went to live with his friend for a while. [77]	Parents feel rejected: The parents are clearly wrestling with the unspoken question: ‘Why, despite all we did for him, did he do this to us?’ They leave their audience in no doubt that he abused them, their love, their trust and their property. [77]
**The experience of incomprehension**	Young people do not feel understood: They do not understand my situation and their scolding is unreasonable. I think they need to love me as they gave birth to me. It seems bad to scold me for no reason. They do not understand me actually. [58]	Young people do not feel understood: They were dissatisfied with their unharmonious family relationships, and criticised their parents for not understanding them, trusting them or listening to them. It made them feel that they were not being valued in their family. [58]
	Parents do not understand their children: We felt so abandoned and let down, didn’t we, because I don’t think as parents we deserved—we hadn’t done anything to deserve that really. [77]	Parents do not understand their children: The suicide makes sense to them when set in the context of a long history of aimlessness and anomie, but the question of why, despite their love and care, he was unable to carve out a meaningful path through life remains largely unresolved. [77]
	Feeling understood plays a role in getting better: Online allows me to have an outlet, an understanding and to share all the weird uncomfortable bits and not be judged—if someone does judge me, I’m fine with it because I don’t feel threatened by online people, they are not in my life, I don’t have to see them every day and to be honest a lot of them can offer a lot of help because they have had similar experiences. [62]	Feeling understood plays a role in getting better: This discourse of empathetic understanding provides website users with two positive and socially valued identities: the understander and the understood. [62]

**Table 9:** Quotations from participants and authors of primary studies to illustrate each theme of superordinate theme 3 (The social and cultural experience).

Themes	Quotations from participants in primary studies	Interpretations of findings offered by authors
**Failure to fit into the group**	The young people do not succeed in meeting social norms: Having a high marriage portion for girls is considered to be socially prestigious: When my family and I went to their house to propose marriage and, during the procedure of wedlock, my father fell in discussion and then quarrelled with my beloved’s uncle on the marriage portion. Finally we had to leave their home. I became so Narahati [sad] for a few months. Last week, again we went to their house. This time they started quarrelling and did not agree to our marriage. [54]	The young people do not succeed in meeting social norms: In addition to interpersonal reasons, social stigma regarding outside marriage relationships appeared as one of the main causes of troubles in love. Both girls were trying to keep their relationship with their boyfriends secret because of strong family opposition. The shame of exposing the relationship was so unbearable for one of the girls that it led her to attempt suicide. [54]
	Parents feel and transmit the emotions associated with this failure: He was having some sort of twitches in his body and was forced to act up in school, to get up and run off, or some such thing, so that no one would see these strange tics, and this was very shameful for him. In the sixth grade, he said ‘‘if I don’t get some help for this, I’m going to kill myself. [76]	Parents feel and transmit the emotions associated with this failure: The parents could tell the boy was ashamed of being too short, twitching his head, arms and legs, or sweating too much […] His suicide note could say that he hated his body. [76]
**Sociocultural facilitators and barriers to suicide and its management**	Religious beliefs protect against suicide: God gave me this life. I cannot take it until he wants to. [52]	Religious beliefs protect against suicide: Leo n (Nicaragua) society traditionally has strict religious norms and values. This is reflected in the view on suicide. [52]
	Religious beliefs can promote suicide: We had good times at the beginning but now all of them became Christians; my mother, my grandma and my nanny so, no matter what I say, all is a sin! If I mention a word, I’ll be punished with hell; the devil will take me far away, and so forth. [52]	Religious beliefs can promote suicide: Some of the informants hinted at the weakening of religious norms and values. Religion sometimes causes conflicts in relationships with parents and relatives. [52]
	The media promote an idealised image of suicide: They have done it to themselves, but they have done it in an very naive state of mind not knowing much and I think they have done it from—well you see things like this on TV a great deal, in soap operas and stuff and that’s what they watch. [82]	The media promote an idealised image of suicide: In a similar way, one view might be that the media today contribute to creating a moral panic around suicidal behaviour in young people by reconstructing it as deviant behavior. [82]
	Stigmatisation limits parents' access to care: Parents whose children’s death certificate says carbon monoxide poisoning or hanging, it is very confronting I suppose, especially if there are younger children involved. Whereas if your child died of cancer it is more acceptable in society’s view—your child dies and otherwise they would still be here. [73]	Stigmatisation limits parents' access to care: The stigma long associated with death, and more specifically to suicide, has affected these parents, leaving them feeling unsure and unable to discuss their child’s death openly. [71]
	The right to die: I do think people have a right over their lives because things are not so clearly defined—there are a lot of grey areas in life and I think there is a difference between committing suicide because they feel there is no hope and not prolonging it because of something else—perhaps some medical reason—they don't want life to be lengthy. . . [42]	The right to die: Both nurses and doctors appeared to share this view relating to the right young people have over their lives, and this coincided with the degree of choice that should be handed over to the individual. [42]
	The taboo of suicide: For the majority of people it is a bad act. For a very few people it is an understandable act—for some of those it's an acceptable act. But for the majority—bad. [42]	The taboo of suicide: They seemed to hold an underlying perception that suicide was wrong and that their child should not have done this. [74]
	The social group protects and supports: I find it to be a very supportive community, and have made several good friends there, and everyone there has something in common, that they find life in general difficulty to do, people there have a work package of empathy and can give great advice and support. [62]	The social group protects and supports: A community member is someone who belongs to a group, is socially integrated, and socially valid. This represents another positive identity for people who use self-harm and suicide websites. Outsiders may lack important knowledge shared by the community and may be unhelpful or even threatening. [62]

### Individual Experience

The three themes belonging to this superordinate theme describe the individual burden and suffering that suicide brings about, albeit in different forms, in all three groups.

#### The experience of distress

The experiences of sadness and of mental or emotional distress are at the heart of the accounts provided by both the youth and the professionals [42–61].

Most of the participants describing their suicidal experience mentioned feelings of depression: sadness [45,50,54,60], sorrow [54], mental pain [49,53,54,56], despair [49,50,54,56,60], detachment [45,56], anger, and irritability [45,49,50,54,60]. The professionals may diagnose depression [44,47,50,55,57,61], but certainly not on a routine basis [42].

The experience of failure is at the heart of this distress [43,44,46–54,56,58,59]: decreased self-esteem [46,49,56], feelings of uselessness [50,52–54,58], incompetence [49], and worthlessness [50,53,54]. Participants sometimes even mentioned self-hatred [48].

Improvement corresponds to exit from the downward spiral of failure [43,46,47,51,59]. The youth interviewed reported the reappearance of positive thoughts [43,47] and restoration of their self-esteem [46,51,59].

#### Self-control

The second subtheme concerns self-control: simultaneously, loss of self-control, impossibility of coping, leading to despair and suicidal action, but also to an attempt to regain control by expressing distress [44–51,53,56,59,61–70].

Many suicidal youth have the impression that they have lost control of their existence [45,49,50,56,63,70], that they can no longer take part in decisions that concern them, no longer influence the course of their own lives [45]. Dealing with their problems, doubts, and fears [44,48,50,61,64,67] or with their painful experiences or strong emotions [44,48,61,68] seems impossible to them. They no longer understand themselves [44,45,56]. Life becomes both hopeless and senseless [50]. Suicide might then appear as a means of regaining self-control [49,56,64].

By talking about themselves, by understanding themselves, changing their point of view, giving a meaning to their existence, these young suicide attempters regain control over their lives [44,46–48,51,53,59,61,62,64–66,68,69]. The therapeutic space [44,46,48,59,64,65,68,69], but also the spaces for individual [51,53] and community [62] expression are settings that enable the young to cope [47,61], where they can rediscover themselves, learn to understand themselves and one another [46,47,61,64,68], change their perspective about things [46,64], give a new meaning to their existence [47,48,61,66], and imagine a positive future [47].

#### Parents’ impotence in the face of the suicide attempters' distress

The family and the treatment teams both reported similar experiences of the distress of these youth, dominated by feelings of helplessness, guilt, anger, and the impression that they are losing control of these young people [71–80].

The family experiences their child’s first suicide attempt in a way resembling the youth’s experience: loss of hope, blame, guilt, self-recrimination, a sense of total failure; rejection, isolation, and incomprehension; powerlessness and helplessness, loss of control [71–76]. The realisation is often sudden, which destabilises the parents still further [71,74]. Whether they were drowning in guilt [71,74,75] or rejecting the responsibility for their child's suffering [77], parents confided their difficulties in rallying round. Their impression of the healthcare system as useless, futile, or rejecting [75,76] reinforces their experience of helplessness.

Healthcare professionals too can feel impotent in the face of the young people's individual feelings of distress: they have the impression that the interventions that they can suggest are not appropriate [78]; those who provide care for these youth wish they had specialised interventions and specific training available [78,79]. They are also helpless in dealing with the parents, whom it might be difficult to help to respond usefully when their attitudes show resistance or shock or when they minimise the risk [80].

### Relational Experience

The three subthemes here describe the importance of the relationship with others at all stages of the suicidal process: i) during the phase when suicidal ideation develops, ii) during the events precipitating the act itself, iii) during the phase of crisis resolution.

#### Changes in the relational distance

At each stage of the suicidal process and its care, the youths, their parents, and the healthcare professionals described movements of rapprochement and of distancing from one another [42,43,45–58,60,61,63,65–72,76,78–84].

The stability of the relationship is important for the youth, as it is for their parents [45,48–50,52–55,57,58,60,61,63,65,67,71,72,76,84]. All of them reported that relational difficulties, separations, mourning, and feelings of insecurity are elements that engender suicidal ideation [45,48,50,52–55,57,58,60,61,63,65,67,84]. Breaches and break-ups, conflicts, separations, losses, and absence can all explain the decision to act [50,52–57,61,63,65,67,72,76,84]. Communication is difficult, and suicide attempts can serve to express one’s distress to the others [45,48–50,52,55–58,60,61,72,76], or to take vengeance on one or more family members or friends [56]. Many professionals also consider that attempted suicide is a mode of communication [42,79,80,82]: according to them, youth use suicide as a powerful form of communication, a way to say something important [82]. The challenge is thus to help the family hear this complex message so that they can work together effectively with the young person [80].

After the suicide attempt, the parents move closer to their child, who becomes a constant preoccupation and sometimes requires constant monitoring.[71,72,76] For the youth, the rapprochement with their families, the reconnection, the improvement of relationships and communication are simultaneously conditions for and consequences of getting better [43,46,47,49,52,56,58,61,66,67,70,81,84]. Relationships with the healthcare provider are central to treatment: the professional must be an unconditional source of love and support [46,51,68,69,83]. Professionals noted that the need for attention is at the heart of treatment: attention from family and friends, but also and especially from healthcare providers, who must commit themselves strongly to the relationship and give of themselves. This type of commitment embarrasses some, who underline the need for particular skills, but also the risk of wasting time, for the result is never certain [42,78–80].

#### Feelings of difference and rejection

Feelings of difference and rejection are present throughout the suicidal process: rejection by peers, family, friends, but also sometimes professionals [45,49–61,65,67,70,71,74,76,77,84].

The feeling of being different from others is very present in the young people’s discourse, shared by the parents [45,49–61,65,67,70,71,76,77,84]. The youth find themselves singular, cannot succeed in resembling their peers, feel isolated and rejected [49,51,54–56,58,59,67,84]. The rejection is based most often on elements of reality: harassment at school, bullying, or discrimination based on sexual orientation [45,50,51,58,61,65]. The youth also say that they find it hard to fit into their family, which is often broken, or in conflict [50,52,55,57,60]. The fear of being judged by others—because of their differences and their inability to adopt the group's common values—amplifies the feeling of solitude and the real isolation [49,52,53,55,57,70]. In some contexts, boys are more vulnerable to isolation than girls [67]. Many parents, reporting what their child has confided in them, confirm these feelings of difference and rejection [71,76,77]. The parents also sometimes feel rejected by their children [71,74,74,76,77].

#### The experience of incomprehension

The experience of incomprehension and of feeling unheard is central to the suicidal process for all the participants: before the suicide attempt, in the precipitating factors, and while in care [42,43,46,47,49–53,55–60,62,65,66,68–72,74,76–80,82,83,85].

The youth do not feel understood [43,49–52,55,57,58,62,66,68,70,83]. The family’s or peer group’s failure to hear leads to the suicidal behaviour [43,49–52,55–58,60,62,66,68,70,83].

The parents, for their part, do not understand [71,72,74,76,77,80]. Faced with the violence of the act, their immediate reaction is denial, distancing from what frightens them [71,74,80], or shock or stupefaction that prevents any reaction [71,80]. Later, they ask themselves numerous questions that remain unanswered [72,74,76,77].

Getting better requires that the youth be understood by those around him or her [43,49,56,58,65], including by empathetic professionals [62,65,66,68], supported by family or a therapeutic setting [46,47,49,51,53,58,59,62,65,66,68–70,83]. Professionals underlined the difficulties of empathy and the contradictory positions, which are a barrier to care [42,78,79,82,85].

### The Social and Cultural Experience

The two subthemes of this superordinate theme describe the socio-cultural dimension of suicide. The peer group, the cultural group, and the society, by the ways they accept or reject youth in distress and their families, play a role in the process of suicidal behaviour and in its management.

#### Failure to fit into the group

Both the youth and their parents underlined the difficulties of belonging to the peer group, to the cultural group, or more broadly, to the social group. Young people associate this failure to fit in with shame, guilt, and anger—and parents often corroborate these feelings [43–45,48–61,63,65–67,70,72,76,77,84].

The self-esteem of young people is based on numerous standards and values—religious, cultural, or of the ideal family structure, school success, beauty, health, or sexuality [43–45,49,50,52–54,57–61,65–67,70,84]. Inability to meet these standards provokes different reactions: shame about the inability to cope and the experience of stigmatisation [44,52–54,56,61,65,67,84], guilt [48,49,52,56,61,67] or anger against others [45,49,50,54]. These emotions can be so strong that young people can consider suicide a conceivable response [50]. Some situations—those of LGBT youth, as well as cultural or religious minorities—are examples of these difficulties [51,55,61,63,65,84].

Parents repeat what their children have said and report this feeling of failure. The shame of what they did or did not do, of their performance in school, of what has happened, of what they cannot accept, of their physical appearance, of who they are or cannot succeed in being [72,76]—all these play a role in the escalation of their distress [77].

#### Sociocultural facilitators and barriers to suicide and its management

The sociocultural environment, religious beliefs, representations of death, community groups: all of these are levers that facilitate or obstacles that block effective care [42,49,51–54,60–63,65–67,69,71–73,77–80,82,83,85].

Religious beliefs are often a protective factor: the principal religions preach that suicide is forbidden, an offense to God, for humans have a duty to take care of themselves [42,52,53,85]. But this ban can also lead to the exclusion of those who are suffering [42,52], and the absence of any consideration of their suffering in their religious beliefs can be "*a reason for engaging in suicidal behaviour*" [42]. In some contexts, this difference in the influence of religion may be more marked according to gender and may protect girls more than boys [67].

Media representations of self-harm behaviour play an important role in suicidal actions [77,82]. According to some healthcare professionals, the media may promote an idealised image of rebellious youth, including those who rebel by suicide [82].

Each group of participants raised the question of the right to die or of whether it is forbidden, an issue underlain by ethical, philosophical, and cultural considerations [42,49,52–54,61,63,65–67,78–80,82,85].

Stigmatisation of distress and of suicidal ideas block access to care for young people and their families [60,67,71–73]. Families that have experienced a child's suicide—attempted or completed—do not allow themselves to talk about it. In particular, in some cultures, suicide is a trauma that affects the entire family and is transmitted from generation to generation [85].

But there are organisations and structures in society that promote care: religious and organisational support for the parents [72] and peer networks for youth [51,53,62,69,83].

## Discussion

This qualitative synthesis of 44 studies questioning youthful suicide attempters, their parents, and their healthcare providers enabled us to identify three superordinate themes which describe their experience: individual experience, relational experience, and social and cultural experience. One result is transversal at the heart of each group’s experience: incomprehension is a barrier to effective care. The violence of the message of a suicidal act and the fears associated with death lead to incomprehension on all sides and interfere with the capacity of both family members and professionals to empathize with the young person.

This incomprehension is present in most of the themes: difficulty in understanding oneself and in coping with one’s individual experience; difference, incomprehension, and rejection in relational experiences; and shame, guilt, and inability to fit into the social or cultural group. The suicidal act, frightening and shocking, reinforces this incomprehension. The family expresses doubts, calls itself into question, but also blames the youth: how could he have done this to us? Professionals cannot make sense of the act. The will to die is unthinkable.

When youth behave suicidally, they impose the violence of their act on others, on family, friends, and on the healthcare providers who support them. The suicide attempt acts out feelings of anger, hatred, and vengeance toward the other: others who are not sufficiently present, did not listen enough, did not understand enough. It is then very hard to be empathetic toward a youth who treats you so aggressively. It is difficult to empathize, identify, with distress that is expressed as violence—violence directed at the family, friends, and professionals. This is the primary observation of this meta-synthesis: everyone experienced and expressed great difficulty in identifying with the distress of these youths. Family members reacted by denial, by distancing themselves from what frightens them. Healthcare providers described their difficulties in being empathetic and asked for assistance, usually framed as a desire for specialised training. The violence addressed to them as the other stupefies their capacity to understand, listen, and empathize.

The response of the sociocultural group to suicidal behaviour is also most often a response to this violence: criminal prosecution for suicidal behaviour, allowed in some countries, is intended to protect the group. The moral condemnation that some participants report, sometimes transmitted between generations, is also intended to limit these actions perceived as aggressive. The results show this to be particularly true in Asia, Africa, and among men in South America [52,54,57,67,84,85]. Religion, when it condemns suicide, protects the group from violence to the detriment of any consideration of the individual's distress. The group is thus protected from the distress linked to loss and mourning, but also and especially from the violence driven by the suicidal behaviour. In the West, finally, the medicalization of suicidal behaviour seeks to give a meaning, labelled in terms of pathology, to this violence [7,43,61].

How can we support a family member whose distress is unthinkable? How can we treat someone when our capacity of empathy is dumbfounded? How can we escape this relation impasse? This is the primary issue in caring for these youths and their families.

## Implications for Practice

It is important to rethink the relationships between doctors, parents, and young patients in the context of attempted suicide. The difficulties of empathy toward these young people interfere both with care and support by their families. The issue in treatment is to witness this violence so that the patient feels understood and heard. The objective of course is to prevent recurrence: when the violence of the message is not heard in treatment, the suicidal potential remains present.

One pathway for envisioning the therapeutic relationship with these patients may be in the concept of intersubjectivity and in the conceptualisation of a "third space" [86–91]. The concept of intersubjectivity envisions the construction of self through the experience of a relationship with another and of interactions with another. The therapeutic relationship can thus be thought of as a place of exchange and construction, as “*two autopoietic human beings with embedded nervous systems that are engaged within a shared environment*, *the intersubjective third space*, *from which new therapeutic possibilities can arise*”[91]. The model of the third space has been envisioned in the care of chronically suicidal patients [87]. It suggests treatment ideas borrowed from the treatment of patients with chronic pain. These situations may promote the appearance of negative empathy based on experiences of hostility, prejudice, and stigmatization [90]. The third space restarts communication in different ways, by sharing the experiences of patients and professionals and makes it possible to combat the professionals' rejection and negative feelings, thus promoting care.

From a relational perspective, the creation of a third space gives the parties the opportunity to create a respectful interpersonal relationship [87]. Professionals must make a commitment to share their representations of patients’ suicide behaviour. The objective here is to establish relative equity within the relationship, so that patients feel understood and able to share their experience [90]. The third space is a staging of the active rapprochement of patients and professionals. The latter share with the patients important representations of the suicidal behaviour and also of the loss of their ability to contain the patient's experience. This rapprochement allows patients to let themselves share this experience.

From social and cultural perspectives, work is necessary on the representations of suicidal behaviour conveyed in the medical world. Numerous disciplines, especially the social sciences, consider suicide, which can thus be envisioned in a collective dimension, as a social fact or as influenced by cultural phenomena [92–96]. The current trend is towards the medicalization of our understanding of suicidal behaviours [7]. Nonetheless, these behaviours are not solely related to medicine and pathology: the psychiatric comorbidities of suicide vary greatly as a function of social, cultural, and educational contexts [7]. Treatment of adolescents with suicidal behaviour and their families should always include multidisciplinary management, including social workers and people with training in education [7,97]. A better understanding and management of suicidal behaviour requires apprehending it in all its psychological, social, and cultural complexity [20].

## Implications for Research

This synthesis has enabled us to examine the perspectives of the principal protagonists of care for young people who take suicidal actions. The parents' perspective has principally focused on the study of families in which those acts were successful. It appears important to develop research about the parents of youth whose attempts failed: what changes does this failed act induce in a family?

The media's fascination with suicide has been studied widely, and youth are particularly exposed.[3,98–100] But media are also powerful tools that enable the circulation of representations. Research must also examine how to use the media as a tool to share representations around suicide.

Finally, more in-depth study is needed of the representations of both the death of a young person and a self-inflicted death, by the integration of the social, anthropological, and philosophical dimensions. The sociological literature on this question (see [95,101,102]) has difficulty associating the individual and societal levels. It is essential to integrate these different perspectives to be able to propose an explanatory model of suicide among the young on which proposals for care can be based.

## Strengths and Limitations of This Review

This review integrates the experience of distress and care of the principal stakeholders participating in the care of youth suicide: suicidal young people, their families, and healthcare professionals. It is based on a rigorous method, tested in medical research [25–27,103–106] and meets the criteria of the principal protocol used in qualitative research (ENTREQ). A systematic review of the principal search engines in this domain enabled us to select a large body of articles. The synthesis is based on the analysis of 44 studies, globally of good quality, published in peer-reviewed journals. The themes proposed here are widely found in the literature. They describe the experience of nearly 900 participants, providing a perspective much larger than any of the initial studies.

The data from the qualitative meta-synthesis includes participants for whom only partial data are available, as well as the interpretations of the researchers whose studies we included. Any generalisation should be cautious. Nonetheless the triangulation of numerous points of view, different methods, and different cultural areas, is a strength that promotes the emergence of theoretical explanatory proposals.

Although the synthesis includes articles from diverse cultural areas, the restriction to articles in either English or French limits the cultural perspectives. For the future of this method, it would be useful to develop methods that would allow the inclusion of data from cultural areas that publish only rarely or even never in English.

The articles included provide only a limited look at the influence of gender on the experience of suicidal behaviour. Nonetheless, other types of studies have explored the role of gender with important results (see for example [107–110]). In the future, qualitative studies on suicidal behaviour should consider its role.

The results of the studies are particularly homogeneous, which in qualitative research is a limitation, for this field seeks to shed light on the question through new perspectives. This observation has already been noted in some meta-syntheses of adult suicide attempters [30,32]. The difficulties related to thinking about death and the message of the suicide may explain this homogeneity. Qualitative research involves the subjective participation of researchers. The question of suicide seems to be difficult for researchers to envision, just as it is for participants to think about.

## Supporting Information

S1 File List of articles excluded in the last step of the review. (DOC)

S1 Table ENTREQ statement. (DOC)

S2 Table Complete search strategy. Performed on July 1, 2013 (updated on May 31, 2014).
(DOC)

S3 Table CASP (Critical Appraisal Skill Program) results. T: Totally met; P: Partially met; N: Not met;?: Unclear.
(DOC)
