# Supplementary material for: Metasynthesis of Youth Suicidal Behaviours: Perspectives of Youth, Parents, and Health Care Professionals
Source: PLoS One. 2015 May 22;10(5):e0127359. doi: 10.1371/journal.pone.0127359 (PMC4441448; doi:10.1371/journal.pone.0127359)
Supplement: S1 File — (DOC) [file pone.0127359.s001.doc]

**File S4. List of the 124 papers excluded in the last step of the review.**

1. **Articles excluded for methodological issues (4)**

Biddle, L., Cooper, J., Owen-Smith, A., Klineberg, E., Bennewith, O., Hawton, K., … Gunnell, D. (2013). Qualitative interviewing with vulnerable populations: Individuals’ experiences of participating in suicide and self-harm based research. Journal of Affective Disorders, 145(3), 356–362. doi:10.1016/j.jad.2012.08.024

Bullock, M., Nadeau, L., & Renaud, J. (2012). Spirituality and religion in youth suicide attempters’ trajectories of mental health service utilization: The year before a suicide attempt. Journal of the Canadian Academy of Child and Adolescent Psychiatry, 21(3), 186–193.

Michel, K., Maltsberger, J. T., Jobes, D. A., Leenaars, A. A., Orbach, I., Stadler, K., … Valach, L. (2002). Discovering the truth in attempted suicide. American Journal of Psychotherapy, 56(3), 424–437.

Wright, J., Briggs, S., & Behringer, J. (2005). Attachment and the body in suicidal adolescents: a pilot study. Clinical Child Psychology & Psychiatry, 10(4), 477–491.

1. **Articles excluded for thematic issues (8)**

Biddle, V., Sekula, L., Zoucha, R., & Puskar, K. (2010). Identification of suicide risk among rural youth: implications for the use of HEADSS. Journal of Pediatric Healthcare, 24(3), 152–167. doi:10.1016/j.pedhc.2009.03.003

Christianson, C. L., & Everall, R. D. (2009). Breaking the silence: school counsellors’ experiences of client suicide. British Journal of Guidance & Counselling, 37(2), 157–168.

Gibson, J., Gallagher, M., & Jenkins, M. (2010). The experiences of parents readjusting to the workplace following the death of a child by suicide. Death Studies, 34(6), 500–528. doi:10.1080/07481187.2010.482879

Gratton, F., & Bouchard, L. (2001). [How adolecents experience the suicide of a friend: an exploratory study.]. Santé Mentale Au Québec, 26(2), 203–226.

Hatchette, J. E., McGrath, P. J., Murray, M., & Finley, G. A. (2008). The role of peer communication in the socialization of adolescents’ pain experiences: a qualitative investigation. BMC Pediatrics, 8(1), 2. doi:10.1186/1471-2431-8-2

Hoffmann, W., Myburgh, C., & Poggenpoel, M. (2010). The lived experiences of late-adolescent female suicide survivors: “a part of me died”. Health SA Gesondheid, 15(1), 36–44. doi:10.4102/hsag.v15i1.493

Nadeem, E., Kataoka, S. H., Chang, V. Y., Vona, P., Wong, M., & Stein, B. D. (2011). The role of teachers in school-based suicide prevention: A qualitative study of school staff perspectives. School Mental Health, 3(4), 209–221.

Slovak, K. (2012). Clinical Concepts in Messaging Strategies to Parents of Depressed and Suicidal Adolescents. Social Work in Mental Health, 10(1), 72–88. doi:10.1080/15332985.2011.625299

1. **Articles excluded for ineligible participants (112)**

Adams, J., Rodham, K., & Gavin, J. (2005). Investigating the “self” in deliberate self-harm. Qualitative health research, 15(10), 1293–1309.

Alexander, M., Haugland, G., Ashenden, P., Knight, E., & Brown, I. (2009). Coping with thoughts of suicide: techniques used by consumers of mental health services. Psychiatric Services, 60(9), 1214–1221.

Babow, I., & Rowe, R. (1990). A suicidal adolescent’s sleeping beauty syndrome: cessation orientations toward dying, sleep, and drugs. Adolescence, 25(100), 791–798.

Balcombe, L., Phillips, L., & Jones, J. (2011). Engagement with Young People Who Self-Harm. Mental Health Practice, 15(2), 14–18.

Band, M., Dein, S., & Loewenthal, K. (2011). Religiosity, coping, and suicidality within the religious Zionist community of Israela thematic qualitative analysis. Mental Health, Religion & Culture, 14(10), 1031–1047.

Di Battista, A., Godfrey, C., Soo, C., Catroppa, C., & Anderson, V. (2014). “In my before life”: relationships, coping and post-traumatic growth in adolescent survivors of a traumatic brain injury. Journal of Rehabilitation Medicine, 46(10), 975–983.

Bell, J., Stanley, N., Mallon, S., & Manthorpe, J. (2010). The role of perfectionism in student suicide: three case studies from the UK. Omega, 61(3), 251–267.

Bergmans, Y., Spence, J. M., Strike, C., Links, P. S., Ball, J. S., Rufo, C., Rhodes, A. E., et al. (2009). Repeat substance-using suicidal clients-how can we be helpful? Social Work In Health Care, 48(4), 420–431.

Berlim MT, Mattevi BS, Pavanello DP, Caldieraro MA, Fleck MPA, Wingate LR, et al. Psychache and suicidality in adult mood disordered outpatients in Brazil. Suicide & life-threatening behavior. 2003;33(3):242–8.

Best, R. (2006). Deliberate self-harm in adolescence: a challenge for schools. British Journal of Guidance & Counselling, 34(2), 161–175.

Biddle, L., Donovan, J., Owen-Smith, A., Potokar, J., Longson, D., Hawton, K., Kapur, N., et al. (2010). Factors influencing the decision to use hanging as a method of suicide: qualitative study. The British Journal Of Psychiatry: The Journal Of Mental Science, 197(4), 320–325.

Biddle, L., Gunnell, D., Owen-Smith, A., Potokar, J., Longson, D., Hawton, K., Kapur, N., et al. (2012). Information sources used by the suicidal to inform choice of method. Journal Of Affective Disorders, 136(3), 702–709.

Biong, S., Karlsson, B., & Svensson, T. (2008). Metaphors of a shifting sense of self in men recovering from substance abuse and suicidal behavior. Journal of Psychosocial Nursing & Mental Health Services, 46(4), 35–41.

Buhnick-Atzil, O., Rubinstein, K., Tuval-Mashiach, R., Fischer, S., Fruchter, E., Large, M., & Weiser, M. (2015). Everyday functioning of male adolescents who later died by suicide: Results of a pilot case-control study using mixed-method analysis. Journal of Affective Disorders, 172, 116–120.

Byrne, S., Morgan, S., Fitzpatrick, C., Boylan, C., Crowley, S., Gahan, H., Howley, J., et al. (2008). Deliberate self-harm in children and adolescents: A qualitative study exploring the needs of parents and carers. Clinical Child Psychology and Psychiatry, 13(4), 493–504.

Chandler MJ, Lalonde CE, Sokol BW, Hallett D. Personal persistence, identity development, and suicide: a study of Native and Non-native North American adolescents. Monographs of the Society for Research in Child Development. 2003;68(2):vii – viii, 1–130; discussion 131–8.

Chesley, K., & Loring-McNulty, N. (2003). Process of suicide: perspective of the suicide attempter. Journal of the American Psychiatric Nurses Association, 9(2), 41–45.

Chowdhury, A. N., Brahma, A., Banerjee, S., & Biswas, M. K. (2007). Media influenced imitative hanging: a report from West Bengal. Indian Journal of Public Health, 51(4), 222–224.

Cleary, M., Jordan, R., Horsfall, J., Mazoudier, P., & Delaney, J. (1999). Suicidal patients and special observation. Journal Of Psychiatric And Mental Health Nursing, 6(6), 461–467.

Coggan, C., Patterson, P., & Fill, J. (1997). Suicide: Qualitative data from focus group interviews with youth. Social Science & Medicine, 45(10), 1563–1570.

Crawford, M. J., Sharpe, D., Rutter, D., & Weaver, T. (2009). Prevention of suicidal behaviour among army personnel: a qualitative study. Journal of the Royal Army Medical Corps, 155(3), 203–207.

Creighton, G., Oliffe, J. L., Butterwick, S., & Saewyc, E. (2013). After the death of a friend: young men’s grief and masculine identities. Social Science & Medicine (1982), 84, 35–43.

Curtis, C. (2006). Sexual abuse and subsequent suicidal behaviour: exacerbating factors and implications for recovery. Journal of Child Sexual Abuse, 15(2), 1–21.

Curtis, C. (2010). Youth perceptions of suicide and help-seeking: “They”d think I was weak or “mental”’. Journal of Youth Studies, 13, 699–715.

DeCou, C. R., Skewes, M. C., & López, E. D. S. (2013). Traditional living and cultural ways as protective factors against suicide: perceptions of Alaska Native university students. International Journal of Circumpolar Health, 72.

Dunleavey, R. (1992). An adequate response to a cry for help? Parasuicide patients’ perceptions of their nursing care. Professional Nurse (London, England), 7(4), 213–215.

Dyregrov K. Assistance from local authorities versus survivors’ needs for support after suicide. Death studies. 2002 Oct;26(8):647–68.

Dyregrov, K. (2009). How do the young suicide survivors wish to be met by psychologists? A user study. Omega, 59(3), 221–238.

Fullagar, S. (2003). Wasted lives - The social dynamics of shame and youth suicide. Journal of Sociology, 39, 291–307.

Fullagar, S., Gilchrist, H., Sullivan, G., & web-support@bath.ac.uk. (2007). The construction of youth suicide as a community issue in urban and regional Australia. Australian e-Journal for the Advancement of Mental Health, 6(2). Retrieved March 5, 2015, from http://www.auseinet.com/journal/vol6iss2/fullagar.pdf

Gaffney, P., Russell, V., Collins, K., Bergin, A., Halligan, P., Carey, C., & Coyle, S. (2009). Impact of patient suicide on front-line staff in Ireland. Death Studies, 33(7), 639–656.

Gelinas, B. L., & Wright, K. D. (2013). The cessation of deliberate self-harm in a university sample: the reasons, barriers, and strategies involved. Archives of Suicide Research: Official Journal of the International Academy for Suicide Research, 17(4), 373–386.

Gilchrist, H., & Sullivan, G. (2006a). Barriers to help-seeking in young people: community beliefs about youth suicide. Australian Social Work, 59(1), 73–85.

Gilchrist, H., & Sullivan, G. (2006b). The role of gender and sexual relations for young people in identity construction and youth suicide. Culture, Health & Sexuality, 8(3), 195–209.

Harris, I. M., & Roberts, L. M. (2013). Exploring the use and effects of deliberate self-harm websites: an Internet-based study. Journal of Medical Internet Research, 15(12), e285.

Harvey, K., & Brown, B. (2012). Health Communication and Psychological Distress: Exploring the Language of Self-harm. Canadian Modern Language Review/ La Revue canadienne des langues vivantes, 68(3), 316–340.

Hill, K., & Dallos, R. (2011). Young people’s stories of self-harm: A narrative study. Clinical Child Psychology and Psychiatry, 17(3), 459–475.

Høifødt, T. S., & Talseth, A.-G. (2006). Dealing with suicidal patients--a challenging task: a qualitative study of young physicians’ experiences. BMC Medical Education, 6, 44–44.

Høifødt, T. S., Talseth, A.-G., & Olstad, R. (2007). A qualitative study of the learning processes in young physicians treating suicidal patients: from insecurity to personal pattern knowledge and self-confidence. BMC Medical Education, 7, 21–21.

Holtman, Z., Shelmerdine, S., London, L., & Flisher, A. (2011). Suicide in a poor rural community in the Western Cape, South Africa: Experiences of five suicide attempters and their families. South African Journal of Psychology, 41(3), 300–309.

Horne, O., & Csipke, E. (2009). From Feeling Too Little and Too Much, to Feeling More and Less? A Nonparadoxical Theory of the Functions of Self-Harm. Qualitative Health Research, 19(5), 655–667.

Ikunaga, A., Nath, S. R., & Skinner, K. A. (2013). Internet suicide in Japan: A qualitative content analysis of a suicide bulletin board. Transcultural Psychiatry, 50(2), 280–302.

Im, M. Y., & Kim, Y. J. (2011). [A phenomenological study of suicide attempts in elders]. Journal of Korean Academy of Nursing, 41(1), 61–71.

Kasckow, J., Appelt, C., Haas, G. L., Huegel, S., Fox, L., Gurklis, J., Zickmund, S., et al. (2012). Development of a recovery manual for suicidal patients with schizophrenia: consumer feedback. Community Mental Health Journal, 48(5), 564–567.

Keyvanara, M., & Haghshenas, A. (2010). The sociocultural contexts of attempting suicide among women in Iran. Health Care for Women International, 31(9), 771–783.

Keyvanara, M., Mousavi, S. G., Malekian, A., & Kianpour, M. (2010). Suicide Prevention: The Experiences of Recurrent Suicide Attempters (A phenomenological study). Iranian Journal of Psychiatry and Behavioral Sciences, 4(1), 4–12.

Kidd, S. A. (2004). “The walls were closing in, and we were trapped” - A qualitative analysis of street youth suicide. Youth & Society, 36, 30–55.

Kizza, D., Hjelmeland, H., Kinyanda, E., & Knizek, B. L. (2012). Alcohol and suicide in postconflict northern Uganda: a qualitative psychological autopsy study. Crisis, 33(2), 95–105.

Kizza, D., Knizek, B., Kinyanda, E., & Hjelmeland, H. (2012). Men in despair: A qualitative psychological autopsy study of suicide in Northern Uganda. Transcultural Psychiatry, 49(5), 696–717.

Knowles, S. E., Townsend, E., & Anderson, M. P. (2012). Youth Justice staff attitudes towards screening for self-harm. Health & Social Care in the Community, 20(5), 506–515.

Kokaliari, E., & Berzoff, J. (2008). Nonsuicidal Self-Injury Among Nonclinical College Women: Lessons From Foucault. Affilia, 23(3), 259–269.

Kraft, T., Jobes, D., Lineberry, T., Conrad, A., & Kung, S. (2010). Brief report: why suicide? Perceptions of suicidal inpatients and reflections of clinical researchers. Archives of Suicide Research, 14(4), 375–382.

Kuipers, P., Appleton, J., & Pridmore, S. (2012). Thematic analysis of key factors associated with Indigenous and non-Indigenous suicide in the Northern Territory, Australia. Rural and Remote Health, 12(4), 2235.

Lakeman, R., & FitzGerald, M. (2008). How people live with or get over being suicidal: a review of qualitative studies. Journal of advanced nursing, 64(2), 114–126.

Lau, U., & van Niekerk, A. (2011). Restorying the self: an exploration of young burn survivors’ narratives of resilience. Qualitative Health Research, 21(9), 1165–1181.

Long, A., & Reid, W. (1996). An exploration of nurses’ attitudes to the nursing care of the suicidal patient in an acute psychiatric ward. Journal of Psychiatric and Mental Health Nursing, 3(1), 29–37.

McAndrew, S., & Warne, T. (2005). Cutting across boundaries: a case study using feminist praxis to understand the meanings of self-harm. International journal of mental health nursing, 14(3), 172–180.

McAndrew, S., & Warne, T. (2010). Coming out to talk about suicide: gay men and suicidality. International Journal Of Mental Health Nursing, 19(2), 92–101.

McAndrew, S., & Warne, T. (2012). Gay children and suicidality: the importance of professional nurturance. Issues In Mental Health Nursing, 33(6), 348–354.

McDermott, E., Roen, K., & Scourfield, J. (2008). Avoiding shame: young LGBT people, homophobia and self-destructive behaviours. Culture, Health & Sexuality, 10(8), 815–829.

McDonald, G., O’Brien, L., & Jackson, D. (2007). Guilt and shame: experiences of parents of self-harming adolescents. Journal of Child Health Care, 11(4), 298–310.

Meeks, S., & Tennyson, K. (2003). Depression, hopelessness, and suicidal ideation in nursing home residents. Journal of Mental Health & Aging, 9(2), 85–96.

Mohanraj, R., Kumar, S., Manikandan, S., Kannaiyan, V., & Vijayakumar, L. (2014). A public health initiative for reducing access to pesticides as a means to committing suicide: Findings from a qualitative study. International Review of Psychiatry, 26(4), 445–452.

Molock, S. D., Barksdale, C., Matlin, S., Puri, R., Cammack, N., & Spann, M. (2007). Qualitative study of suicidality and help-seeking behaviors in African American adolescents. American Journal Of Community Psychology, 40(1-2), 52–63.

Mugisha, J., Hjelmeland, H., Kinyanda, E., & Knizek, B. L. (2011). Distancing: a traditional mechanism of dealing with suicide among the Baganda, Uganda. Transcultural Psychiatry, 48(5), 624–642.

Mugisha, J., Hjelmeland, H., Kinyanda, E., & Knizek, B. L. (2013). Religious views on suicide among the Baganda, Uganda: a qualitative study. Death Studies, 37(4), 343–361.

Neto, M. L. R., de Almeida, J. C., Reis, A. O. A., & de Abreu, L. C. (2012). Narratives of suicide. Healthmed, 6, 3565–3570.

Oldershaw, A., Richards, C., Simic, M., & Schmidt, U. (2008). Parents’ perspectives on adolescent self-harm: qualitative study. The British Journal of Psychiatry, 193(2), 140–144.

Olson, L. M., Wahab, S., Thompson, C. W., & Durrant, L. (2011). Suicide notes among Native Americans, Hispanics, and Anglos. Qualitative Health Research, 21(11), 1484–1494.

Owens, C., Lambert, H., Donovan, J., & Lloyd, K. R. (2005). A qualitative study of help seeking and primary care consultation prior to suicide. The British Journal Of General Practice: The Journal Of The Royal College Of General Practitioners, 55(516), 503–509.

Owens, C., Owen, G., Belam, J., Lloyd, K., Rapport, F., Donovan, J., & Lambert, H. (2011). Recognising and responding to suicidal crisis within family and social networks: qualitative study. BMJ (Clinical Research Ed.), 343, d5801–d5801.

Owens, C., Owen, G., Lambert, H., Donovan, J., Belam, J., Rapport, F., & Lloyd, K. (2009). Public involvement in suicide prevention: understanding and strengthening lay responses to distress. BMC public health, 9, 308.

Proulx, M., & Gratton, F. (2006). The process followed by helping peers during their experience with suicidal teenagers: an exploratory study [French]. Sante Mentale au Quebec, 31(1), 145–168.

Raphael, H., Clarke, G., & Kumar, S. (2006). Exploring parents’ responses to their child’s deliberate self-harm. Health Education, 106(1), 9–20.

Ratnarajah, D., Maple, M., & Minichiello, V. (2014). Understanding family member suicide narratives by investigating family history. Omega, 69(1), 41–57.

Ratnayake, R., & Links, P. (2009). Examining student perspectives on suicidal behaviour and its prevention in Sri Lanka. International Journal of Social Psychiatry, 55(5), 387–400.

Rezaie, L., Hosseini, S. A., Rassafiani, M., Najafi, F., Shakeri, J., & Khankeh, H. R. (2014). Why self-immolation? A qualitative exploration of the motives for attempting suicide by self-immolation. Burns: Journal of the International Society for Burn Injuries, 40(2), 319–327.

Rissanen, M.-L., Kylmä, J., & Laukkanen, E. (2008). Descriptions Of Self-Mutilation Among Finnish Adolescents: A Qualitative Descriptive Inquiry. Issues in Mental Health Nursing, 29(2), 145–163.

Rissanen, M.-L., Kylmä, J., & Laukkanen, E. (2009a). Helping adolescents who self-mutilate: parental descriptions. Journal of Clinical Nursing, 18(12), 1711–1721.

Rissanen, M.-L., Kylmä, J., & Laukkanen, E. (2009b). Descriptions of Help by Finnish Adolescents Who Self-Mutilate. Journal of Child and Adolescent Psychiatric Nursing, 22(1), 7–15.

Rissanen, M.-L., Kylmä, J., & Laukkanen, E. (2011). Self-mutilation among Finnish adolescents: Nurses’ conceptions. International Journal of Nursing Practice, 17(2), 158–165.

Rissanen, M.-L., Kylma, J., & Laukkanen, E. (2012). Helping Self-Mutilating Adolescents: Descriptions of Finnish Nurses. Issues in Mental Health Nursing, 33(4), 251–262.

Rissanen, M.-L., Kylmä, J. P. O., & Laukkanen, E. R. (2008). Parental conceptions of self-mutilation among Finnish adolescents. Journal of Psychiatric and Mental Health Nursing, 15(3), 212–218.

Rivlin, A., Ferris, R., Marzano, L., Fazel, S., & Hawton, K. (2013). A typology of male prisoners making near-lethal suicide attempts. Crisis, 34(5), 335–347.

Roen, K., Scourfield, J., & McDermott, E. (2008). Making sense of suicide: a discourse analysis of young people’s talk about suicidal subjecthood. Social Science & Medicine (1982), 67(12), 2089–2097.

Ron, P. (2004). Depression, Hopelessness, and Suicidal Ideation Among the Elderly: A Comparison Between Men and Women Living in Nursing Homes and in the Community. Journal of Gerontological Social Work, 43(2-3), 97–116.

Samuelsson, M., Wiklander, M., Asberg, M., & Saveman, B. I. (2000). Psychiatric care as seen by the attempted suicide patient. Journal Of Advanced Nursing, 32(3), 635–643.

Sandy, P. T. (2013). Motives for self-harm: views of nurses in a secure unit. International Nursing Review, 60(3), 358–365.

Schoppmann, S., Schröck, R., Schnepp, W., & Büscher, A. (2007). “Then I just showed her my arms . . .” Bodily sensations in moments of alienation related to self-injurious behaviour. A hermeneutic phenomenological study. Journal of psychiatric and mental health nursing, 14(6), 587–597.

Schwartz, K., Pyle, S., Dowd, M., & Sheehan, K. (2010). Attitudes and beliefs of adolescents and parents regarding adolescent suicide. Pediatrics, 125(2), 221–227.

Scourfield, J., Jacob, N., Smalley, N., Prior, L., & Greenland, K. (2007). Young people’s gendered interpretations of suicide and attempted suicide. Child & Family Social Work, 12(3), 248–257.

Scourfield, J., Roen, K., & McDermott, L. (2008). Lesbian, gay, bisexual and transgender young people’s experiences of distress: Resilience, ambivalence and self-destructive behaviour. Health & Social Care in the Community, 16(3), 329–336.

Slovak, K., & Singer, J. (2012). Engaging parents of suicidal youth in a rural environment. Child & Family Social Work, 17(2), 212–221.

De Stefano, J., Atkins, S., Noble, R. N., & Heath, N. (2012). Am I competent enough to be doing this?: A qualitative study of trainees’ experiences working with clients who self-injure. Counselling Psychology Quarterly, 25(3), 289–305.

Stegemann, T., Brüggemann-Etchart, A., Badorrek-Hinkelmann, A., & Romer, G. (2010). [The function of music in the context of non-suicidal self injury]. Praxis Der Kinderpsychologie Und Kinderpsychiatrie, 59(10), 810–830.

Straiton, M., Roen, K., Dieserud, G., & Hjelmeland, H. (2013). Pushing the boundaries: understanding self-harm in a non-clinical population. Archives of Psychiatric Nursing, 27(2), 78–83.

Strickland, C. J., & Cooper, M. (2011). Getting into trouble: perspectives on stress and suicide prevention among Pacific Northwest Indian youth. Journal Of Transcultural Nursing: Official Journal Of The Transcultural Nursing Society / Transcultural Nursing Society, 22(3), 240–247.

Strickland, C. J., Walsh, E., & Cooper, M. (2006). Healing fractured families: parents’ and elders’ perspectives on the impact of colonization and youth suicide prevention in a pacific northwest American Indian tribe. Journal Of Transcultural Nursing: Official Journal Of The Transcultural Nursing Society / Transcultural Nursing Society, 17(1), 5–12.

Sun, F.-K., Long, A., Boore, J., & Tsao, L.-I. (2005). Nursing people who are suicidal on psychiatric wards in Taiwan: action/interaction strategies. Journal Of Psychiatric And Mental Health Nursing, 12(3), 275–282.

Sun, F.-K., Long, A., Boore, J., & Tsao, L.-I. (2006). Patients and nurses’ perceptions of ward environmental factors and support systems in the care of suicidal patients. Journal Of Clinical Nursing, 15(1), 83–92.

Sun, F., Long, A., Boore, J., & Tsao, L. (2006). A theory for the nursing care of patients at risk of suicide. Journal of Advanced Nursing, 53(6), 680–690.

Taylor, T. L., Hawton, K., Fortune, S., & Kapur, N. (2009). Attitudes towards clinical services among people who self-harm: systematic review. The British Journal Of Psychiatry: The Journal Of Mental Science, 194(2), 104–110.

Travasso, S. M., Rajaraman, D., & Heymann, S. J. (2014). A qualitative study of factors affecting mental health amongst low-income working mothers in Bangalore, India. BMC women’s health, 14, 22.

Valach, L., Michel, K., Dey, P., & Young, R. (2006). Linking Life- and Suicide-Related Goal Directed Processes: A Qualitative Study. Journal of Mental Health Counseling, 28(4), 353–372.

Wexler, L. (2009). Identifying colonial discourses in Inupiat young people’s narratives as a way to understand the no future of Inupiat youth suicide. American Indian and Alaska Native Mental Health Research, 16(1), 1–24.

Wexler, L. M. (2006). Inupiat youth suicide and culture loss: Changing community conversations for prevention. Social Science & Medicine (1982), 63(11), 2938–2948.

Whitlock, J., Pietrusza, C., & Purington, A. (2013). Young adult respondent experiences of disclosing self-injury, suicide-related behavior, and psychological distress in a web-based survey. Archives of Suicide Research: Official Journal of the International Academy for Suicide Research, 17(1), 20–32.

Wiklander, M., Samuelsson, M., & Åsberg, M. (2003). Shame reactions after suicide attempt. Scandinavian Journal of Caring Sciences, 17(3), 293–300.

Wolk-Wasserman, D. (1985). The intensive care unit and the suicide attempt patient. Acta Psychiatrica Scandinavica, 71(6), 581–595.

Wood, L., Byram, V., Gosling, A. S., & Stokes, J. (2012). Continuing bonds after suicide bereavement in childhood. Death Studies, 36(10), 873–898.

Yi, & Hwang. (2011). Feminist phenomenology on the life experiences of young Korean women with attempted suicide. Korean Journal of Adult Nursing, 23(2), 123–134.

Remafedi G. Suicidality in a venue-based sample of young men who have sex with men. J Adolesc Health. 2002 Oct;31(4):305–10.
