# Supplementary material for: Metasynthesis of Youth Suicidal Behaviours: Perspectives of Youth, Parents, and Health Care Professionals
Source: PLoS One. 2015 May 22;10(5):e0127359. doi: 10.1371/journal.pone.0127359 (PMC4441448; doi:10.1371/journal.pone.0127359)
Supplement: S2 Table — Performed on July 1, 2013 (updated on May 31, 2014). (DOC) [file pone.0127359.s003.doc]

**Table S2. Complete search strategy.** Performed on July 1, 2013 (updated on May 31, 2014).

| **Medline (Pubmed) (1990-2013, English) (Updated 2013-May 2014)** |
| --- |
| ((MH “Suicide+”) OR (MH “Suicidal Ideation”) OR (MH “Suicide, Attempted”) OR (“suicide Attempts”) OR (“suicide”) OR (“attempted suicide”) OR (“suicidal ideation”) OR (“suicide ideation”) OR (“suicidal behaviour”) OR (“youth suicide”) OR (MH “Self mutilation”) OR (MH “Self-Injurious Behavior+”) OR (“overdose”) OR (“self poison*”) OR (“self inflict*”) OR (“self harm*”) OR (“self cut*”) OR (“self destruct*”) OR (“self-injur*”) OR (“self mutilate*”)) AND ((MH “Adolescent”) OR (MH “Young Adult”) OR (MH “Adolescent Psychology”) OR (MH “Adolescent Psychiatry”) OR (MH “Adolescent Behavior”) OR (MH “Adolescent Development”) OR (“teenagers”) OR (“teens”) OR (“adolescence”) OR (“adolescent”) OR (“adolescents”) OR (“young adult”) OR (“young”)) AND ((MH “Qualitative research”) OR (MH “Nursing Methodology Research”) OR (MH “Focus Groups”) OR (MH “Observation”) OR (“qualitative research”) OR (“qualitative study”) OR (“qualitative method”)) AND ((MH “Knowledge”)OR (MH “Psychology”) OR (MH “Self Concept”) OR (MH “Adolescent Psychiatry”) OR (MH “Attitude”) OR (MH “Perception”) OR (MH “Self Concept”) OR (“perception”) OR (“attitude”) OR (“feeling”) OR (“knowledge”) OR (“belief”) OR (“view”) OR (“perspective”) OR (“opinion”) OR (“experience”) OR (“image”) OR (“self concept”) OR (“barrier*”) OR (“psycholog*”) OR (“psychiatry”))  **[194 results]** |
| **PsycINFO (1990-2013, English) (Updated 2013-May 2014)** |
| ((DE “Suicide+”) OR (DE “Attempted Suicide”) OR (DE “Suicidal Ideation”) OR (“suicide Attempts”) OR (“suicide”) OR (“attempted suicide”) OR (“suicidal ideation”) OR (“suicide ideation”) OR (“suicidal behaviour”) OR (“youth suicide”) OR (DE “Self mutilation”) OR (DE “Self Injurious Behavior”) OR (DE “Self Destructive Behavior”) OR (“overdose”) OR (“self poison*”) OR (“self inflict*”) OR (“self harm*”) OR (“self cut*”) OR (“self destruct*”) OR (“self-injur*”) OR (“self mutilate*”)) AND ((DE “Adolescent Psychiatry”) OR (DE “Adolescent Psychology”) OR (DE “Adolescent Psychopathology”) OR (DE “Adolescent Psychotherapy+”) OR (DE “Adolescent Attitudes”) OR (DE “Adolescent Development”) OR (“teenagers”) OR (“teens”) OR (“adolescence”) OR (“adolescent”) OR (“adolescents”) OR (“young adult”) OR (“young”)) AND ((DE "Qualitative Research") OR (DE "Interviews") OR (DE "Intake Interview") OR (DE "Interview Schedules") OR (DE "Psycho diagnostic Interview") OR (DE "Grounded Theory") OR (DE "Observation Methods") OR (DE "Ethnography") OR (DE "Discourse Analysis") OR (DE "Content Analysis") OR (DE "Phenomenology") OR (DE "Philosophies") OR (DE "Constructivism") OR (DE "Hermeneutics") OR (DE "Narratives") OR (DE "Biography") OR (DE "Life Review") OR (DE "Storytelling") OR (“qualitative research”) OR (“qualitative study”) OR (“qualitative method”)) AND ((DE "Attitudes") OR (DE "Knowledge (General)") OR (DE "Psychology") OR (“perception”) OR (“attitude”) OR (“feeling”) OR (“knowledge”) OR (“belief”) OR (“view”) OR (“perspective”) OR (“opinion”) OR (“experience”) OR (“image”) OR (“self concept”) OR (“barrier*”) OR (“psycholog*”) OR (“psychiatry”))  **[169 results]** |
| **CINAHL Plus - Cumulative Index to Nursing and Allied Health Literature (EBSCO Publishing) (1990-2013, English) (Updated 2013-May 2014)** |
| ((MH “Suicide+”) OR (MH “Suicide, Attempted”) OR (MH “Suicidal Ideation”) OR (“suicide Attempts”) OR (“suicide”) OR (“attempted suicide”) OR (“suicidal ideation”) OR (“suicide ideation”) OR (“suicidal behaviour”) OR (“youth suicide”) OR (“overdose”) OR (“self poison*”) OR (“self inflict*”) OR (“self harm*”) OR (“self cut*”) OR (“self destruct*”) OR (“self-injur*”) OR (“self mutilate*”) OR (MH “overdose”) OR (MH “Self-Injurious Behavior”) OR (MH ”Injuries, Self-Inflicted”)) AND ((MH “Adolescence+”) OR (MH “Adolescent Care”) OR (MH “Adolescent Health”) OR (MH “Adolescent Psychiatry”) OR (MH “Adolescent Psychology”) OR (MH “Adolescent Development”) OR (MH “Adolescent Behavior”) OR (“teenagers”) OR (“teens”) OR (“adolescence”) OR (“adolescent”) OR (“adolescents”) OR (“young adult”) OR (“young”)) AND ((MH "Qualitative Studies+") OR (MH "Focus Groups") OR (MH "Interviews+") OR (MH "Narratives") OR (MH "Observational Methods+") OR (MH "Discourse Analysis") OR (MH "Thematic Analysis") OR (MH "Semantic Analysis") OR (MH "Field Studies") OR (MH "Audiorecording") OR (MH "Constant Comparative Method") OR (MH "Content Analysis") OR (MH "Field Notes") OR (“qualitative research”) OR (“qualitative study”) OR (“qualitative method”)) AND ((MH "Attitude+") OR (MH "Knowledge+") OR (MH "Self Concept+") OR (MH "Psychology+") OR (“perception”) OR (“attitude”) OR (“feeling”) OR (“knowledge”) OR (“belief”) OR (“view”) OR (“perspective”) OR (“opinion”) OR (“experience”) OR (“image”) OR (“self concept”) OR (“barrier*”) OR (“psycholog*”) OR (“psychiatry”))  **[593 results]** |
| **Embase (Ovid) (1990-2013, English) (Updated 2013-May 2014)** |
| (‘suicide'/exp OR ‘suicide attempt'/exp OR ‘suicidal behavior'/exp OR ‘suicidal ideation'/exp OR (suicide attempts) OR (suicide) OR (attempted suicide) OR (suicidal ideation) OR (suicide ideation) OR (suicidal behaviour) OR (youth suicide) OR ‘automutilation’/exp OR (self mutilat*) OR (overdose) OR (self poison*) OR (self inflict*) OR (self harm*) OR (self cut*) OR (self destruct*) OR (self injur*)) AND (‘adolescent'/exp OR ‘child behavior'/exp OR ‘adolescent development'/exp OR ‘adolescent disease'/exp OR ‘adolescent health'/exp OR ‘child psychiatry'/exp OR ‘child psychology'/exp OR (teenagers) OR (teens) OR (adolescence) OR (adolescent) OR (adolescents) OR (young adult) OR (young)) AND ('qualitative research'/exp OR 'narrative'/exp OR 'observational study'/exp OR 'thematic analysis'/exp OR 'content analysis'/exp OR 'constant comparative method'/exp OR (qualitative research) OR (qualitative study) OR (qualitative method)) AND ('attitude'/exp OR 'knowledge'/exp OR 'psychology'/exp OR 'self concept'/exp OR (perception) OR (attitude) OR (feeling) OR (knowledge) OR (belief) OR (view) OR (perspective) OR (opinion) OR (experience) OR (image) OR (self concept) OR (barrier*) OR (psycholog*) OR (psychiatry))  **[266 results]** |
| **SSCI – Social Sciences Citation Index (1990-2013, English) (Updated 2013-May 2014)** |
| (“suicide attempts” OR “suicide” OR “attempted suicide” OR “suicidal ideation” OR “suicide ideation” OR “suicidal behaviour” OR “youth suicide” OR “Self mutilate*” OR “Self Injur*” OR “overdose” OR “self poison*” OR “self inflict*” OR “self harm*” OR “self cut*” OR “self destruct*”) AND (“teenagers” OR “teens” OR “adolescence” OR “adolescent” OR “adolescents” OR “young adult” OR “young”) AND (“case study” OR “constant comparative” OR “content analysis” OR “descriptive study” OR “discourse analysis” OR “ethnography” OR “ethnographic” OR “Focus group” OR "focus groups" OR “grounded theory” OR “interview*” OR “narrative*” OR “observation*” OR “qualitative method*” OR “qualitative research” OR “qualitative study” OR “thematic analysis” OR “semi-structured” OR “in depth”) AND (“Perception” OR “Attitude” OR “Feeling” OR “Knowledge” OR “Belief” OR “View” OR “Perspective” OR “Opinion” OR “Experience” OR “Image” OR "self concept" OR “barrier*” OR “psycholog*”)  **[582 results]** |
