# Supplementary material for: Metasynthesis of Youth Suicidal Behaviours: Perspectives of Youth, Parents, and Health Care Professionals
Source: PLoS One. 2015 May 22;10(5):e0127359. doi: 10.1371/journal.pone.0127359 (PMC4441448; doi:10.1371/journal.pone.0127359)
Supplement: S3 Table — T: Totally met; P: Partially met; N: Not met;?: Unclear. (DOC) [file pone.0127359.s004.doc]

**Table S3. CASP (Critical Appraisal Skill Program) results.**

***Legend: T: Totally met; P: Partially met; N: Not met; ?: Unclear***

| CASP | | Anderson et Al. (2000) (1) | Anderson et Al. (2003)(2) | Anderson et Al. (2005)(3) | Anderson et al. (2012)(4) | Bennet et Al. (2003)(5) | Baker et Fortune (2008)(6) | Beekrum et al. (2011)(7) | Bennett et al (2002)(8) | Bergmans et al. (2009)(9) | Bostik et Everall (2006)(10) | Bostik et Everall (2007)(11) | Daly et Al. (2005)(12) | Diamond et al. (2011)(13) | Everall (2000)(14) | Everall et al. (2005)(15) | Everall et Bostik (2006) (16) | Everall et Altrows (2006) (17) | Fenaughty et Harré (2003)(18) | Greidanus et Everall (2010)(19) | Herrera et al. (2006)(20) | Jo et al. (2011)(21) | Jordan et al. (2012)(22) | Keynavara et al. (2011)(23) |
| --- | --- | --- | --- | --- | --- | --- | --- | --- | --- | --- | --- | --- | --- | --- | --- | --- | --- | --- | --- | --- | --- | --- | --- | --- |
| 1 | Was there a clear statement of the aims of the research? | T | T | T | T | T | T | T | T | T | T | T | T | T | T | T | T | T | T | T | T | T | T | P |
| 2 | Is a qualitative methodology appropriate? | T | T | T | T | T | T | T | T | T | T | T | T | T | T | T | T | T | T | P | T | P | T | T |
| 3 | Was the research design appropriate to address the aims of the research? | T | T | T | T | T | T | T | T | T | T | T | T | T | T | P | T | T | T | P | T | P | T | T |
| 4 | Was the recruitment strategy appropriate to the aims of the research? | P | P | T | T | T | P | T | T | T | T | T | T | T | T | P | T | P | T | N | T | P | T | P |
| 5 | Were the data collected in a way that addressed the research issue? | T | T | T | T | N | P | T | T | T | T | T | T | T | T | T | T | T | T | T | T | P | T | T |
| 6 | Has the relationship between researcher and participants been adequately considered? | T | T | P | T | T | P | N | N | T | T | T | N | T | T | N | T | T | N | N | T | P | T | N |
| 7 | Have ethical issues been taken into consideration? | N | N | N | T | T | T | T | N | T | T | T | T | T | P | T | T | T | T | T | T | T | T | T |
| 8 | Was the data analysis sufficiently rigorous? | T | T | P | P | P | P | P | P | T | T | T | T | T | T | N | T | P | P | T | T | P | T | N |
| 9 | Is there a clear statement of findings? | T | T | T | T | P | T | N | P | T | T | T | P | T | T | N | T | N | P | N | T | P | T | N |
| 10 | How valuable is the research? | T | T | T | T | P | T | T | P | P | T | T | P | T | T | T | T | P | T | P | T | P | T | P |

***Legend: T: Totally met; P: Partially met; N: Not met; ?: Unclear***

| CASP | | Maple et Al. (2007)(24) | Maple et Al. (2010)(25) | Medina et Luna (2006)(26) | Medina et al. (2011)(27) | Lindqvist et al. (2008)(28) | Nirui et Al. (1999)(29) | Osafo et Al. (2011)(30) | Orri et Al. (2014)(31) | Owens et Al. (2008)(32) | Paulson et Everall (2003)(33) | Rodham et Al. (2007)(34) | Shilubane et al. (2012)(35) | Sinclair et Al. (2005)(36) | Slovak et Al. (2012)(37) | Tallaksen et Al. (2013)(38) | Törnblom et Al. (2013)(39) | Sun et Hui (2007)(40) | Walsh et al. (1997)(41) | Wasserman et al. (2008)(42) | Yang (2012)(43) | Zayas et al. (2010)(44) |
| --- | --- | --- | --- | --- | --- | --- | --- | --- | --- | --- | --- | --- | --- | --- | --- | --- | --- | --- | --- | --- | --- | --- |
| 1 | Was there a clear statement of the aims of the research? | T | P | T | T | T | T | T | T | T | T | T | T | T | T | T | T | T | T | T | T | P |
| 2 | Is a qualitative methodology appropriate? | T | T | T | T | T | T | T | T | T | T | T | P | T | T | T | T | T | T | T | T | T |
| 3 | Was the research design appropriate to address the aims of the research? | T | T | T | T | T | T | T | T | T | T | T | T | T | T | T | T | T | P | T | T | P |
| 4 | Was the recruitment strategy appropriate to the aims of the research? | P | N | T | T | T | T | T | T | P | T | T | P | T | T | T | N | T | T | T | T | T |
| 5 | Were the data collected in a way that addressed the research issue? | T | P | T | T | T | T | T | T | P | T | T | P | T | T | T | T | T | T | T | T | P |
| 6 | Has the relationship between researcher and participants been adequately considered? | P | N | T | T | T | P | T | T | P | T | T | N | P | P | T | T | T | T | P | T | P |
| 7 | Have ethical issues been taken into consideration? | N | T | T | T | T | T | T | T | T | T | T | T | T | T | T | T | T | T | N | T | N |
| 8 | Was the data analysis sufficiently rigorous? | T | N | T | P | T | P | T | T | P | P | T | N | P | T | T | T | T | T | P | T | P |
| 9 | Is there a clear statement of findings? | T | N | T | P | T | T | T | T | T | T | T | N | P | P | T | T | T | T | P | T | T |
| 10 | How valuable is the research? | T | P | T | T | P | T | T | T | P | P | T | P | P | T | T | T | T | T | P | T | T |

1. Anderson M, Standen PJ, Nazir S, Noon JP. Nurses’ and doctors’ attitudes towards suicidal behaviour in young people. Int J Nurs Stud. févr 2000;37(1):1‑11.

2. Anderson M, Standen PJ, Noon JP. Nurses’ and doctors’ perceptions of young people who engage in suicidal behaviour: a contemporary grounded theory analysis. Int J Nurs Stud. août 2003;40(6):587‑597.

3. Anderson M, Standen PJ, Noon JP. A Social Semiotic Interpretation of Suicidal Behaviour in Young People. J Health Psychol. mai 2005;10(3):317‑331.

4. Anderson J, Hurst M, Marques A, Millar D, Moya S, Pover L, et al. Understanding suicidal behaviour in young people referred to specialist CAMHS: A qualitative psychoanalytic clinical research project. J Child Psychother. 2012;38(2):130‑153.

5. Bennett S, Coggan C, Adams P. Problematising depression: young people, mental health and suicidal behaviours. Soc Sci Med 1982. juill 2003;57(2):289‑299.

6. Baker D, Fortune S. Understanding self-harm and suicide websites: A qualitative interview study of young adult website users. Crisis J Crisis Interv Suicide Prev. 2008;29(3):118‑122.

7. Beekrum R, Valjee SR, Collings SJ. An emic perspective on the dynamics of non-fatal suicidal behaviour in a sample of South African Indian women. South Afr J Psychol. mars 2011;41:63‑73.

8. Bennett S, Coggan C, Adams P. Young People’s Pathways to Well-being Following a Suicide Attempt. Int J Ment Health Promot. août 2002;4(3):25‑32.

9. Bergmans Y, Langley J, Links P, Lavery JV. The perspectives of young adults on recovery from repeated suicide-related behavior. Crisis. 2009;30(3):120‑127.

10. Bostik KE, Everall RD. In My Mind I Was Alone: Suicidal Adolescents’ Perceptions of Attachment Relationships. Int J Adv Couns. sept 2006;28(3):269‑287.

11. Bostik KE, Everall RD. Healing from suicide: adolescent perceptions of attachment relationships. Br J Guid Couns. févr 2007;35(1):79‑96.

12. Daly P. Mothers living with suicidal adolescents: a phenomenological study of their experiences. J Psychosoc Nurs Ment Health Serv. mars 2005;43(3):22.

13. Diamond GM, Shilo G, Jurgensen E, D’Augelli A, Samarova V, White K. How depressed and suicidal sexual minority adolescents understand the causes of their distress. J Gay Lesbian Ment Health. mars 2011;15(2):130‑151.

14. Everall RD. The meaning of suicide attempts by young adults. Can J Couns. avr 2000;34(2):111‑125.

15. Everall RD, Bostik KE, Paulson BL. I’m sick of being me: developmental themes in a suicidal adolescent. Adolescence. déc 2005;40(160):693‑708.

16. Everall RD, Bostik KE, Paulson BL. Being in the Safety Zone: Emotional Experiences of Suicidal Adolescents and Emerging Adults. J Adolesc Res. 1 juill 2006;21(4):370‑392.

17. Everall RD, Altrows KJ, Paulson BL. Creating a Future: A Study of Resilience in Suicidal Female Adolescents. J Couns Dev. oct 2006;84(4):461‑470.

18. Fenaughty J, Harré N. Life on the Seesaw: A Qualitative Study of Suicide Resiliency Factors for Young Gay Men. J Homosex. 2003;45(1):1‑22.

19. Greidanus E, Everall RD. Helper therapy in an online suicide prevention community. Br J Guid Couns. mai 2010;38(2):191‑204.

20. Herrera A, Dahlblom K, Dahlgren L, Kullgren G. Pathways to suicidal behaviour among adolescent girls in Nicaragua. Soc Sci Med. févr 2006;62:805‑814.

21. Jo K-H, An G, Sohn K-C. Qualitative content analysis of suicidal ideation in Korean college students. Collegian. juin 2011;18(2):87‑92.

22. Jordan J, McKenna H, Keeney S, Cutcliffe J, Stevenson C, Slater P, et al. Providing meaningful care: learning from the experiences of suicidal young men. Qual Health Res. 2012;22(9):1207‑1219.

23. Keyvanara M, Haghshenas A. Sociocultural contexts of attempting suicide among Iranian youth: a qualitative study. East Mediterr Health J Rev Santé Méditerranée Orient Al-Majallah Al-Ṣiḥḥīyah Li-Sharq Al-Mutawassiṭ. 2011;17(6):529‑535.

24. Maple M, Plummer D, Edwards H, Minichiello V. The effects of preparedness for suicide following the death of a young adult child. Suicide Life Threat Behav. avr 2007;37(2):127‑134.

25. Maple M, Edwards H, Plummer D, Minichiello V. Silenced voices: Hearing the stories of parents bereaved through the suicide death of a young adult child. Health Soc Care Community. mai 2010;18(3):241‑248.

26. Medina C, Luna G. Suicide attempts among adolescent Mexican American students enrolled in special education classes. Adolescence. juin 2006;41(162):299‑312.

27. Medina CMO, Dahlblom K, Herrera A, Kullgren G. I Keep My Problems to Myself: Pathways to Suicide Attempts in Nicaraguan Young Men. Suicidol Online. 2011;2:17‑28.

28. Lindqvist P, Johansson L, Karlsson U. In the aftermath of teenage suicide: a qualitative study of the psychosocial consequences for the surviving family members. BMC Psychiatry. 21 avr 2008;8:26‑26.

29. Nirui M, Chenoweth L. The response of healthcare services to people at risk of suicide: a qualitative study. Aust N Z J Psychiatry. juin 1999;33(3):361‑371.

30. Osafo J, Hjelmeland H, Akotia CS, Knizek BL. The meanings of suicidal behaviour to psychology students in Ghana: A qualitative approach. Transcult Psychiatry. nov 2011;48:643‑659.

31. Orri M, Paduanello M, Lachal J, Falissard B, Sibeoni J, Revah-Levy A. Qualitative Approach to Attempted Suicide by Adolescents and Young Adults: The (Neglected) Role of Revenge. Harris F, éditeur. PLoS ONE. 6 mai 2014;9(5):e96716.

32. Owens C, Lambert H, Lloyd K, Donovan J. Tales of biographical disintegration: how parents make sense of their sons’ suicides. Sociol Health Illn. mars 2008;30(2):237‑254.

33. Paulson BL, Everall RD. Suicidal Adolescents: Helpful Aspects of Psychotherapy. Arch Suicide Res. oct 2003;7(4):309‑321.

34. Rodham K, Gavin J, Miles M. I hear, I listen and I care: a qualitative investigation into the function of a self-harm message board. Suicide Life Threat Behav. août 2007;37(4):422‑430.

35. Shilubane HN, Ruiter RAC, Bos AER, van den Borne B, James S, Reddy PS. Psychosocial determinants of suicide attempts among black South African adolescents: a qualitative analysis. J Youth Stud. 2012;15:177‑189.

36. Sinclair J. Understanding resolution of deliberate self harm: qualitative interview study of patients’ experiences. BMJ. 14 mai 2005;330(7500):1112‑0.

37. Slovak K, Singer J. Engaging parents of suicidal youth in a rural environment. Child Fam Soc Work. mai 2012;17(2):212‑221.

38. Tallaksen DW, Bråten K, Tveiten S. « … You are not particularly helpful as a helper when you are helpless » A qualitative study of public health nurses and their professional competence related to suicidal adolescents. Vard Nord Utveckl Forsk. 2013;33(1):46‑50.

39. Törnblom AW, Werbart A, Rydelius P-A. Shame behind the masks: the parents’ perspective on their sons’ suicide. Arch Suicide Res Off J Int Acad Suicide Res. 2013;17(3):242‑261.

40. Sun R, Hui E. Building social support for adolescents with suicidal ideation: implications for school guidance and counselling. Br J Guid Couns. août 2007;35(3):299‑316.

41. Walsh SM, Minor-Schork D. Suicidal adolescent perceptions after an art future image intervention. Appl Nurs Res ANR. 1997;10(1):19‑26.

42. Wasserman D, Tran Thi Thanh H, Pham Thi Minh D, Goldstein M, Nordenskiöld A, Wasserman C. Suicidal process, suicidal communication and psychosocial situation of young suicide attempters in a rural Vietnamese community. World Psychiatry Off J World Psychiatr Assoc WPA. févr 2008;7(1):47‑53.

43. Yang S. A Life History of a Korean Adolescent Girl Who Attempted Suicide. Death Stud. mars 2012;36(3):253‑269.

44. Zayas L, Gulbas L, Fedoravicius N, Cabassa L. Patterns of distress, precipitating events, and reflections on suicide attempts by young Latinas. Soc Sci Med. juin 2010;70(11):1773‑1779.
